# The gut microbiome enhances breast cancer immunotherapy following bariatric surgery

**DOI:** 10.1172/jci.insight.187683

**Published:** 2025-04-24

**Authors:** Margaret S. Bohm, Sydney C. Joseph, Laura M. Sipe, Minjeong Kim, Cameron T. Leathem, Tahliyah S. Mims, Nathaniel B. Willis, Ubaid A. Tanveer, Joel H. Elasy, Emily W. Grey, Madeline E. Pye, Zeid T. Mustafa, Barbara Anne Harper, Logan G. McGrath, Deidre Daria, Brenda Landvoigt Schmitt, Jelissa A. Myers, Patricia Pantoja Newman, Brandt D. Pence, Marie Van der Merwe, Matthew J. Davis, Joseph F. Pierre, Liza Makowski

**Affiliations:** 1 Department of Microbiology, Immunology, and Biochemistry, College of Medicine, University of Tennessee Health Science Center (UTHSC), Memphis, Tennessee, USA.; 2 Department of Medicine, Division of Hematology and Oncology, College of Medicine, UTHSC, Memphis, Tennessee, USA.; 3 Department of Biology, University of Mary Washington, Fredericksburg, Virginia, USA.; 4 Department of Nutritional Sciences, College of Agricultural and Life Sciences, University of Wisconsin–Madison, Madison, Wisconsin, USA.; 5 Flow and Cell Sorting Core, Office for Vice Chancellor of Research, UTHSC, Memphis, Tennessee, USA.; 6 College of Health Sciences, University of Memphis, Memphis, Tennessee, USA.; 7 Department of Surgery, and; 8 UTHSC Center for Cancer Research, College of Medicine, UTHSC, Memphis, Tennessee, USA.

**Keywords:** Immunology, Microbiology, Oncology, Breast cancer, Cancer immunotherapy, Obesity

## Abstract

Bariatric surgery is associated with improved breast cancer (BC) outcomes, including greater immunotherapy effectiveness in a preclinical BC model. A potential mechanism of bariatric surgery–associated protection is the gut microbiota. Here, we demonstrate the dependency of improved immunotherapy response on the post–bariatric surgery gut microbiome via fecal microbiota transplantation (FMT). Response to αPD-1 immunotherapy was significantly improved following FMT from formerly obese bariatric surgery–treated mice. When stool from post–bariatric surgery patients was transplanted into recipient mice and compared to the patients’ presurgery transplants, postsurgery microbes significantly reduced tumor burden and doubled immunotherapy effectiveness. Microbes impact tumor burden through microbially derived metabolites, including branched-chain amino acids (BCAAs). Circulating BCAAs correlated significantly with natural killer T (NKT) cell content in the tumor microenvironment in donor mice after bariatric surgery and FMT recipients of donor cecal content after bariatric surgery compared with obese controls. BCAA supplementation replicated improved αPD-1 effectiveness in 2 BC models, supporting the role of BCAAs in increased immunotherapy effectiveness after bariatric surgery. Ex vivo exposure increased primary NKT cell expression of antitumor cytokines, demonstrating direct activation of NKT cells by BCAAs. Together, the findings suggest that reinvigorating antitumor immunity may depend on bariatric surgery–associated microbially derived metabolites, namely BCAAs.

## Abstract 2

Bariatric surgery causes long term changes to our gut microbiome, which we demonstrated could improve the body’s immune response to current breast cancer treatments.

## Graphical abstract

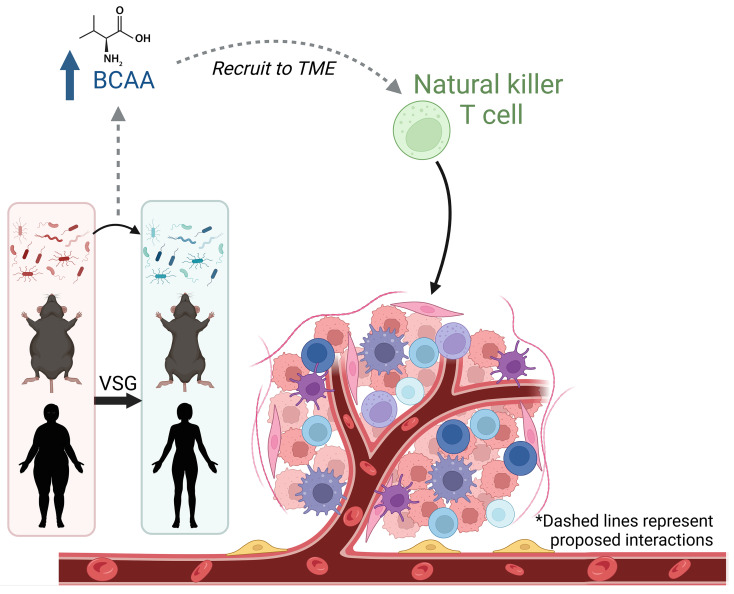

## Introduction

Breast cancer (BC) is the leading cause of cancer-related death among females worldwide (1). Patients with a history of metabolic weight loss surgery, or bariatric surgery, have reduced risk of cancer onset, as well as improved responses to immunotherapy, lower stage of severity at diagnosis, longer time to recurrence, and decreased mortality upon subsequent BC diagnosis (2–11). However, mechanisms for these beneficial outcomes remain unknown.

Bariatric surgery has been increasing in prevalence throughout the past decades due to recent advances in laparoscopic approaches for obese patients (12, 13). Retrospective studies demonstrate that bariatric surgery, including both vertical sleeve gastrectomy (VSG) and Roux-en-Y gastric bypass, is associated with improved BC outcomes up to 10 years after surgery (3, 4, 14). Estrogen receptor–negative BC shows the most significant decrease in cancer-related mortality regardless of the amount of weight lost (15). We have reported that VSG in a preclinical obese model significantly reduced BC tumor progression and improved response to immunotherapy (16), but the specific mechanisms behind the cancer protection after VSG remain undefined.

One potential mechanism for bariatric surgery–induced improvements in tumor progression and superior response to therapy is changes to the microbiome and its associated metabolites. Obesity is associated with dysbiosis of the gut microbiome characterized by decreased microbial diversity and increased abundance of both classically pathogenic species and short-chain fatty acid–producing (SCFA-producing) bacteria (17, 18). Composition of the gut microbiome is altered in patients after bariatric surgery (19–21). These changes persist beyond the initial weight loss period of 12 months (19). Bariatric surgery in both patients and preclinical models results in an intermediate microbiome phenotype with notably increased microbial diversity, although surgery does not result in a complete return to a lean gut microbiome composition (21–23). Previous work has demonstrated that fecal microbiota transplantation (FMT) of microbes from mice with previous bariatric surgery into nonoperated, germ-free mice induces weight loss (24), indicating that the gut microbiome plays a role in the beneficial impacts of bariatric surgery. Importantly, clinical studies demonstrate that the gut microbiome impacts the onset, progression, and response to chemo- and immunotherapy in certain types of cancer, including extraintestinal cancers such as BC (25–31). FMTs are currently under active investigation for their potential therapeutic uses in cancer treatment (32). The gut microbiome potentially acts through producing or modifying microbially derived metabolites such as SCFAs, amino acids, and bile acids, which can impact antitumor immunity (33, 34).

In this study, we examined whether the benefits of bariatric surgery depend on the microbiome and are potentially transferrable to improve BC outcomes in FMT recipients. Utilizing microbes isolated from a preclinical model after VSG that was established to lead to reduced tumor burden (16), we performed FMTs into lean recipient mice to determine impacts on BC progression and response to αPD-1 immune checkpoint blockade (ICB). We found that FMT of cecal contents from mice with previous VSG improved the response to αPD-1 ICB. Furthermore, when stool from patients with post–bariatric surgery weight loss was transplanted into recipient mice and compared to patient stool collected before bariatric surgery, the impact of the FMT resulted in significantly blunted tumor burden and improved ICB response. Our findings suggested that the improved response to ICB is mediated by an increase in circulating microbially derived branched-chain amino acids (BCAAs) unique to FMT with VSG microbes and elevated natural killer (NK)/NKT cells. Supplementation with BCAAs elevated NKT cells and demonstrated consistent improvement in ICB efficacy in 2 BC models. Lastly, primary invariant NKT (iNKT) cells treated with BCAAs ex vivo demonstrated activated antitumor phenotypes. Taken together, these findings suggest a potential mechanism by which bariatric surgery–associated microbially derived metabolites improve ICB effectiveness, which may be readily translated to patients.

## Results

### Microbes transplanted from VSG donors did not alter body weight or composition.

**Figure 1:**
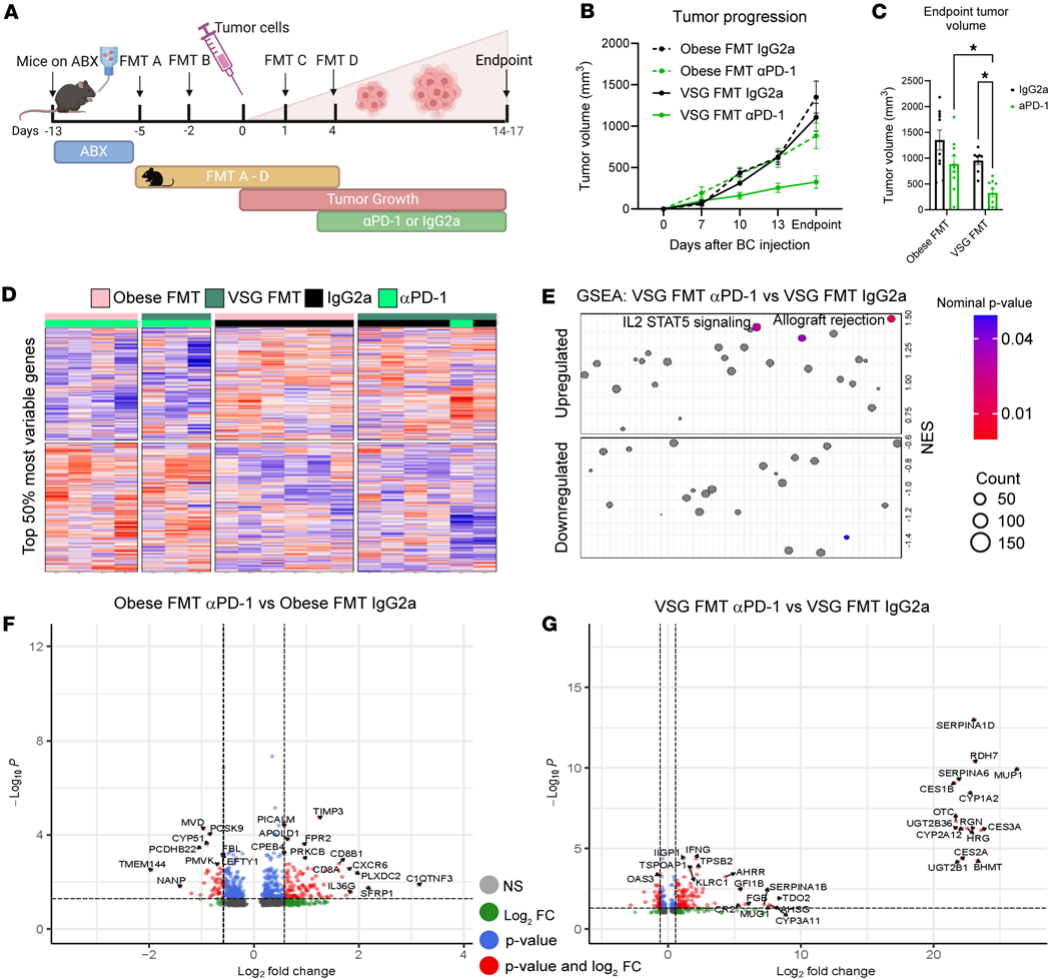
Microbes transplanted from VSG donors improved response to αPD-1 immune checkpoint blockade therapy. (**A**) Study schema: Female C57BL/6J mice were purchased from The Jackson Laboratory at 4 weeks of age. On day –13, a broad-spectrum antibiotic cocktail (1 g/L neomycin, 1 g/L ampicillin, 1 g/L cefoperazone, and 0.5 g/L vancomycin) was administered for 8 days via drinking water to ablate the commensal gut microbiome prior to fecal microbiota transplantation (FMT). Following 2 FMT gavages (FMT A and B), E0771 breast cancer cells were injected into the fourth right mammary fat pad and monitored for 3 weeks. Two additional FMT gavages occurred following cell injection (FMT C and D). αPD-1 immune checkpoint blockade (ICB) therapy or IgG2a isotype control was administered at 200 μg/mouse intraperitoneally every 3 days from tumor injection to endpoint. Additional controls not depicted in the cartoon are reported in Supplemental Figure 1. (**B**) Tumor progression was recorded by digital caliper and quantified until endpoint. *n* = 8–10 per group. (**C**) Tumor volume at endpoint presented as mean ± SEM with comparisons determined by 2-way ANOVA. *n* = 8–10 per group. (**D**) Semisupervised heatmap of RNA-seq transcriptomic analysis of tumors from mice receiving Obese or VSG FMT and αPD-1 ICB or IgG2a isotype control. Top 50% of differentially expressed genes are shown. *n* = 3–6 per group. (**E**) Dot plots of gene set enrichment analysis (GSEA) results representing upregulated and downregulated HALLMARK pathways in VSG FMT recipients plotted as αPD-1 ICB versus IgG2a isotype control, with significant nominal *P* values denoted by dot color and gene count signified by dot size. Pathways are ranked by normalized enrichment score (NES). (**F** and **G**) Volcano plots of significant differentially expressed genes in tumors from Obese FMT (**F**) and VSG FMT (**G**) recipients plotted as αPD-1 ICB versus IgG2a isotype control with log_2_ fold change (FC) of greater than 1.5 and adjusted *P* value of less than 0.05. *n* = 3–6 per group. *P < 0.05.

Our group has previously published that VSG slows tumor progression and improves response to αPD-L1 ICB treatment (16). To investigate the dependence of improved immunotherapy efficacy after bariatric surgery on the gut microbiome, a preclinical FMT study was conducted as shown in the study schema (Figure 1A) using various murine controls or cecal contents from formerly obese bariatric surgery donors (VSG FMT) and donors that remained obese after sham surgery that served as controls (Obese FMT). Female C57BL/6J mice were purchased from The Jackson Laboratory at 4 weeks of age and placed on low-fat diet (LFD) containing 10% kcal from fat to establish baseline weights. Prior to FMT, a cocktail of broad-spectrum antibiotics (ABX) composed of vancomycin, neomycin, ampicillin, and cefoperazone was used to ablate the commensal gut microbiome of all mice, while ABX-free control mice (ABX–/FMT–) remained on plain autoclaved water. Water intake was measured daily during the 8-day ABX intervention period. The ABX cocktail was well tolerated and did not impact water intake when compared to control cages that did not receive ABX (Supplemental Figure 1A and Supplemental Figure 2A; supplemental material available online with this article; https://doi.org/10.1172/jci.insight.187683DS1). Following 8 days of ABX treatment, the mice were transferred back to plain autoclaved water to cease ABX exposure. After a 6-hour washout period on plain water, the first FMT dose of donor cecal contents was administered by oral gavage, with control groups receiving no gavage. FMTs were administered every 3 days for a total of 4 FMT doses. E0771 BC cell injections occurred following the first 2 FMTs (Figure 1A). ICB was injected intraperitoneally every 3 days, starting when tumors first became palpable 3 days after injection (Figure 1A). Control mice included mice without ABX exposure or FMT gavages (ABX–/FMT–), mice receiving ABX but not FMT (ABX+/FMT–), and mice receiving microbes from lean donors (Lean FMT). FMTs, ICB, and cancer progression did not affect body weight (Supplemental Figure 1B and Supplemental Figure 2B). At endpoint, multiple tissues relevant to obesity, tumor, or immunobiology were isolated, including adipose tissue (fat depots) such as mesenteric, brown, mammary gland, and gonadal. No changes in adipose weights were observed among control groups (Supplemental Figure 1C). While mesenteric and brown adipose did not demonstrate a change in mass after FMT or ICB, mammary gland and gonadal adipose demonstrated significantly increased weights in VSG recipients only after αPD-1 immunotherapy (Supplemental Figure 2, C–F). Spleen and liver mass was not altered by FMT or ICB (Supplemental Figure 1D and Supplemental Figure 2, G and H, respectively).

### Microbes transplanted from VSG donors increased immunotherapy effectiveness in recipient mice.

Tumor progression was quantified for 2.5 weeks prior to endpoint (Figure 1, A–C, Supplemental Figure 1, E and F, and Supplemental Figure 2, I and J). Control mice without ABX exposure and in the absence of FMT gavages (ABX–/FMT–) or mice receiving just ABX but not FMT (ABX+/FMT–) displayed identical tumor progression (Supplemental Figure 1E), similar to our previous publications (16, 35–37). Lean FMT recipients had moderately slowed tumor progression when compared with controls (Supplemental Figure 1, E and F), while mice receiving microbes from donors that lost weight via caloric restriction had no change in tumor progression compared to Obese FMT (data not shown). VSG FMT recipients did not significantly reduce tumor progression compared to Obese FMT recipients in the IgG2a controls (Figure 1B and Supplemental Figure 2, K and M). With αPD-1 ICB intervention, mice receiving Obese FMT displayed only a moderate and insignificant reduction in tumor progression and endpoint volumes compared with IgG2a controls (Figure 1, B and C, and Supplemental Figure 2, K and L). Strikingly, VSG FMT recipients displayed a potently improved response to αPD-1 ICB, with significantly reduced tumor progression observed as early as day 10 after cell injection (Figure 1, B and C, and Supplemental Figure 2, M and N). Endpoint tumor volumes showed a substantial 71% reduction in volume after ICB in the VSG FMT recipients compared with a 35% reduction in Obese FMT recipient controls, a 2-fold improvement in αPD-1 effectiveness comparing response of Obese versus VSG donor FMTs (Figure 1C). These results demonstrate an impact of microbes on immunotherapy efficacy unique to mice receiving microbes from donors after VSG.

### Microbes transplanted from VSG donors and treated with immunotherapy demonstrated IL-2/STAT5 antitumor pathway activation.

Transcriptomic analysis of fresh frozen tumors harvested at endpoint was undertaken to reveal potential pathways mediating reduced tumor burden after VSG FMT and immunotherapy. Comparing the top 50% most variable genes using semisupervised clustering, heatmap representation demonstrates that the unbiased mapping was driven by IgG2a or αPD-1 ICB treatment, with the surgery not demonstrating dramatic dichotomy between the Obese FMT or the VSG FMT groups (Figure 1D).

We next examined the gene sets enriched in 50 HALLMARK pathways (Figure 1E and Supplemental Figure 3, A–C) and individual gene variation (Figure 1, F and G, and Supplemental Figure 3, D and E). When comparing the response to surgical intervention or ICB, genes that were greater than 1.5-fold changed with an adjusted *P* value of less than 0.05 were noted and used for subsequent pathway analysis. When comparing the impact of the FMT in the IgG2a control groups, 34 differentially expressed genes (DEGs) were regulated overall in tumors, with 4 genes upregulated and 30 downregulated by VSG FMT compared with the Obese FMT group (Supplemental Figure 3E). Gene set enrichment analysis (GSEA) HALLMARK pathway analysis indicated that 3 pathways were significantly downregulated by VSG FMT in recipient tumors compared with Obese FMT recipient tumors receiving only IgG2a control ICB: HALLMARK_Allograft_Rejection, HALLMARK_Complement, and HALLMARK_IL2_STAT5_signaling (Supplemental Figure 3A). Analysis of response to ICB in obese controls included 39 DEGs significantly modified, with 24 genes upregulated and 15 genes downregulated by αPD-1 ICB compared with IgG2a control therapy in Obese FMT tumors (Figure 1F). GSEA results demonstrate that the HALLMARK pathways significantly upregulated in tumors from Obese FMT αPD-1–treated group compared with IgG2a-treated controls include HALLMARK_Glycolysis, HALLMARK_Hypoxia, HALLMARK_Reactive oxygen species (ROS) pathway, HALLMARK_mTORC1, and HALLMARK_UV response (Supplemental Figure 3B).

The most dramatic response was present in mice with VSG FMT after ICB, which were mice with the smallest tumors, with 159 genes demonstrated to be significantly altered by αPD-1 ICB relative to IgG2a control intervention, where 126 genes were upregulated and 33 were downregulated in the tumors (Figure 1G). As expected, genes in both the Obese and VSG group include immune-related genes responsive to αPD-1 ICB, including elevated CD8 subunit α (*Cd8a*), CD8 subunit β1 (*Cd8b1*), CD274 antigen (*Cd274*) or PD-L1, interferon-inducible (IFN-inducible) GTPase 1 (*Iigp1*), and killer cell lectin-like receptor subfamily C, member 1 (*Klrc1*), which together support a potently activated antitumor immune response (Figure 1, F and G). Unique responses to ICB detected only in the VSG FMT recipients include expected upregulation of immune-related genes such as IFN-γ (*Ifng*), which would contribute to this group having the smallest tumor burden. Interestingly, some of the genes with over 20 log_2_ fold upregulation in response to αPD-1 ICB in the VSG FMT tumors are unique and not found in the Obese FMT comparison. These genes include cytochrome P450, family 1, subfamily A, polypeptide 2 (*Cyp1a2*), which is involved in hydrogen peroxide synthesis, histidine-rich glycoprotein (*Hrg*), which positively regulates the immune response to tumor cells, several genes in the steroid or eicosanoid pathway including cytochrome P450, family 2, subfamily A, polypeptide 12 (*Cyp2a12*), and retinol dehydrogenase 7(*Rdh7*), and genes involved in amino acid biosynthesis of arginine from ornithine via ornithine transcarbamylase (*Otc*) or tryptophan catabolism via tryptophan 2,3-dioxygenase (*Tdo2*). Downregulated genes include metabolic enzymes pyruvate dehydrogenase kinase, isoenzyme 4 (*Pdk4*), and acetyl-Coenzyme A acetyltransferase 2 (*Acat2*) (Figure 1G). GSEA analysis revealed that in VSG FMT recipient mice, those treated with αPD-1 ICB significantly upregulated immune-related pathways HALLMARK_Allograft_Rejection and HALLMARK_IL2_STAT5_signaling (Figure 1E), which will contribute to a robust immune response (38).

### Protection after bariatric surgery is potent after transplantation of patient pre- and postsurgery stool.

**Figure 2:**
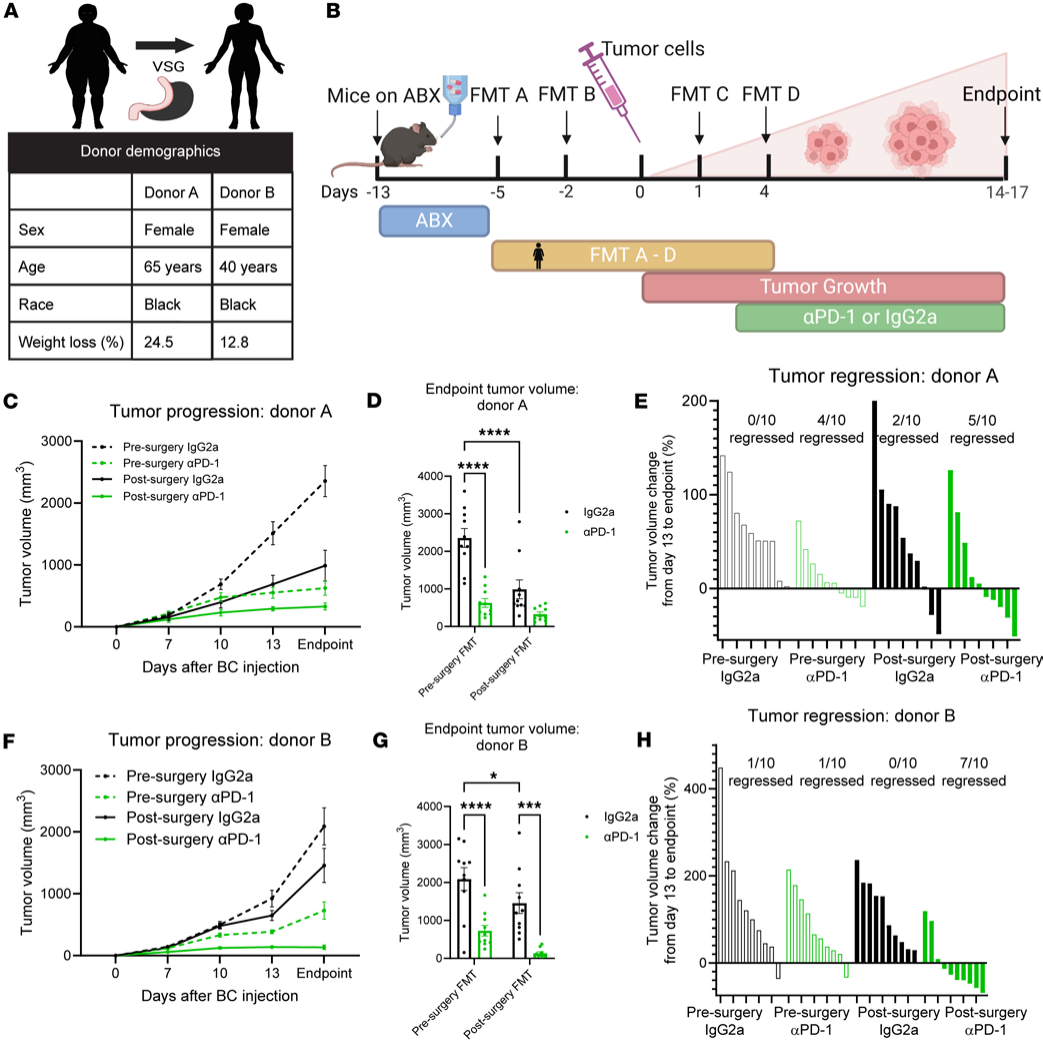
Microbes from patients after bariatric surgery reduced tumor burden and improved response to immunotherapy in recipient mice. (**A**) FMT gavages were derived from stool collected from patients before bariatric surgery or 6 months after bariatric surgery transplanted into separate recipient groups. (**B**) Study schema is identical to Figure 1A with the exception that FMT samples were from patient stool. (**C**) Tumor progression following FMT from Donor A was recorded by digital caliper. *n* = 8–10 recipients per donor group. (**D**) Tumor volume at endpoint following FMT from Donor A presented as mean ± SEM with comparisons determined by 2-way ANOVA. *n* = 8–10 recipients per donor group. (**E**) Tumor regression following FMT from Donor A presented as percentage change in tumor volume is shown as rank-sorted treatment groups. The number of mice with regressed tumors is shown out of the total. (**F**) Tumor progression following FMT from Donor B was recorded by digital caliper. *n* = 10 recipients per donor group. (**G**) Tumor volume at endpoint following FMT from Donor B presented as mean ± SEM with comparisons determined by 2-way ANOVA. *n* = 10 recipients per donor group. (**H**) Tumor regression following FMT from Donor B presented as percentage change in tumor volume is shown as rank-sorted treatment groups. The number of mice with regressed tumors is shown out of the total. *P < 0.05; ***P < 0.001; ****P < 0.0001.

To demonstrate the translational relevance of the microbiota in BC progression and response to immunotherapy, patient fecal samples were collected prior to bariatric surgery (presurgery time point) and 6 months after bariatric surgery (postsurgery time point) following sustained weight losses of 25% and 13%, respectively (Figure 2A). We performed FMT as described in Figure 1A but used patient fecal samples instead of murine fecal samples (Figure 2B). Also, for the patient FMT study, female C57BL/6J mice were placed on a Mediterranean diet to provide the optimal environment for human microbiome engraftment. As above, ABX did not impact water intake (Supplemental Figure 4A and Supplemental Figure 5A). FMTs did not affect body weight, adipose tissue weights, or organ (spleen and liver) weights of recipient mice, as mice remained lean throughout the study (Supplemental Figure 4, B–H and Supplemental Figure 5, B–H). ICB treatment resulted in a minor but significant increase in some adipose weights (Supplemental Figure 4C and Supplemental Figure 5, C–E).

Analysis of tumor volumes over time demonstrated that mice receiving patient fecal content after bariatric surgery displayed significantly reduced tumor progression compared with presurgery donor content transplanted in IgG2a controls (Figure 2, C and F). Tumor endpoint volumes were significantly reduced by more than half in mice receiving postsurgery FMT compared with presurgery FMT from Donor A in the IgG2a controls (Figure 2, C–E). The impact of bariatric surgery alone comparing pre- and postsurgery FMT from Donor B was also significant, with a 25% reduction in tumor volume in the IgG2a groups (Figure 2, F–H).

**Figure 3:**
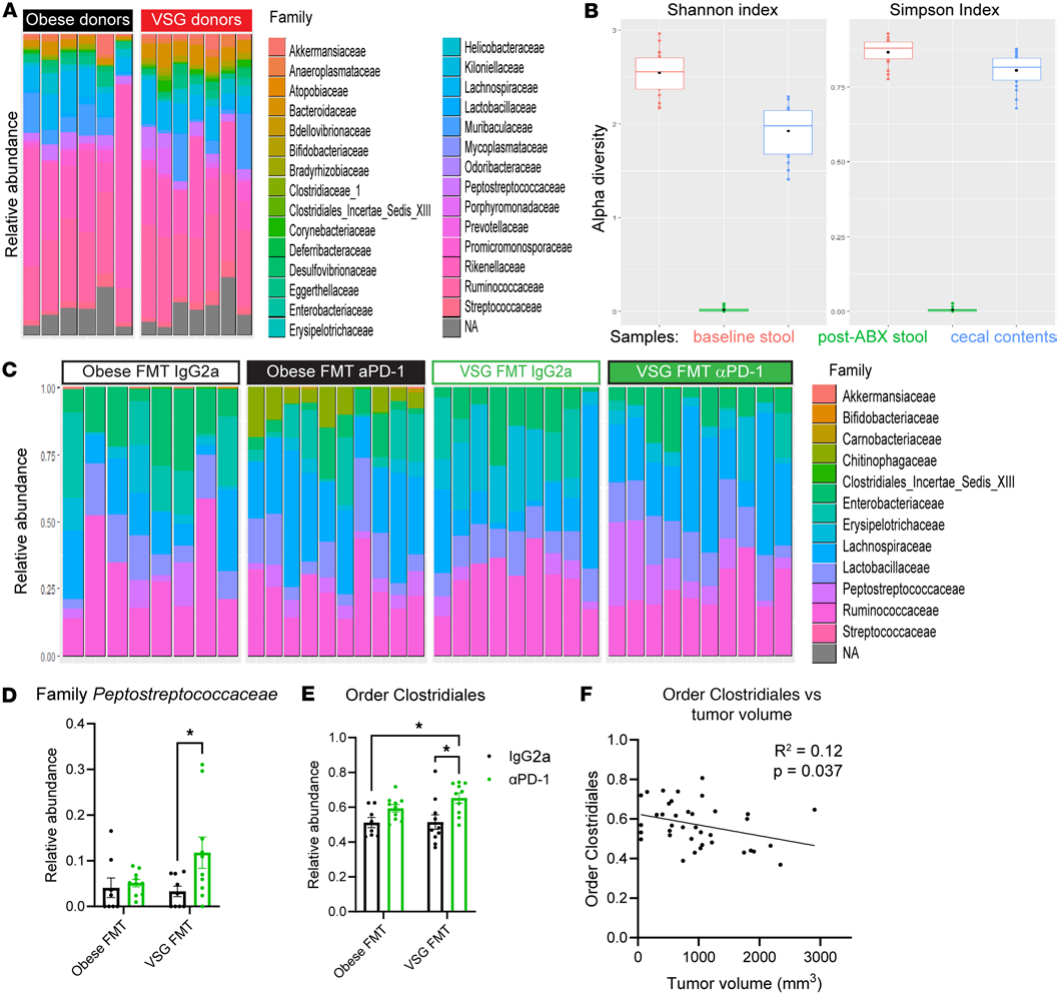
Microbes transplanted from VSG donors suggest elevated order Clostridiales in response to αPD-1 immune checkpoint blockade therapy. (**A**) Relative abundance of microbial families is reported from donor cecal contents harvested from obese sham or VSG donors at endpoint. Relative abundance was calculated using the top 1000 taxa identified using phyloseq (https://www.bioconductor.org/packages/release/bioc/html/phyloseq.html). *n* = 6–7 per group. (**B**) The α diversity in recipients across the timeline of the study included baseline stool collected on day –13, postantibiotic stool collected on day –5, and cecal contents taken at endpoint. Shannon and Simpson indices were calculated using phyloseq. *n* = 8–10 per group for VSG FMT recipients. Boxes represent the interquartile range (IQR) between the first and third quartiles, and the horizontal line inside the box defines the median. Whiskers represent the range of values from highest to lowest. Black dots inside the box represent the mean. (**C**) Relative abundance of microbial families is reported in recipient cecal contents harvested at FMT study endpoint. *n* = 8–10 per group. (**D** and **E**) Differential abundance analysis of endpoint cecal contents for family *Peptostreptococcaceae* (**D**) and order Clostridiales (**E**). Significant comparisons between relative abundance values were determined by 2-way ANOVA. *n* = 9–10 per group. *P < 0.05. (**F**) Abundance of order Clostridiales members in the cecal contents at endpoint were correlated with endpoint tumor volume via MaAsLin2 analysis. *R*^2^ = 0.12, *P* = 0.037, *n* = 38.

Similar to mouse-to-mouse FMT (Figure 1), we next tested the effectiveness of ICB in both Donor A and Donor B FMT studies. In mice receiving αPD-1, there was a significant antitumor response demonstrating reduced progression in presurgery FMT recipients (Figure 2, C–E and F–H). The combination of ICB plus postsurgery FMT content demonstrated potently blunted tumor progression and reduced endpoint volumes, with significant reduction in Donor B (Figure 2, C–E, and Figure 3, F–H). The most remarkable response in the combination therapy using post–bariatric surgery FMT plus ICB was the regression of tumors that were palpable but shrunk in response to therapy and became impalpable. In Donor A, 50% of the tumors regressed (5 of 10 tumors), with an additional 2 minimally progressing from day 13 of tumor growth to endpoint (Figure 2E). In Donor B, the combination therapy was even more potent, with 70% (7 of 10 tumors) demonstrating regression, with an additional tumor minimally progressing (Figure 2H). The groups graphed separately for each donor and group highlight the progression of individual tumors throughout the course of the experiment and the effects of the combination therapy (Supplemental Figure 4, I–L, and Supplemental Figure 5, I–L). Thus, mice receiving microbes from patients after surgery had a significantly reduced tumor progression, demonstrating that the gut microbiome associated with bariatric surgery after transplantation per se is effective in reducing tumor burden and inducing tumor regression. Furthermore, the combination therapy of post–bariatric surgery microbiome plus αPD-1 led to robust regression compared with presurgery recipients even with ICB.

### Microbes from VSG and Obese donors were successfully transplanted into recipient mice.

To confirm the efficacy of both the ABX cocktail and FMT experimental strategy (32), stool samples and endpoint cecal contents were collected for 16S sequencing from FMT donors and recipients from the mouse-to-mouse FMT study. Comparison of family-level abundance of endpoint cecal contents from the Obese or VSG donor mice (16) displayed minor variability in the microbiome, with the greatest increase detected in family *Muribaculaceae* (Figure 3A). Prior to the introduction of ABX or any other intervention (“Baseline Stool”), recipient mice displayed diverse microbiomes with high α diversity by both Shannon and Simpson indices (Figure 3B). Following 8 days of ABX treatment, stool samples were collected (“Post-ABX Stool”) and demonstrated that the ABX cocktail was successful in ablating the commensal gut microbiome, as shown by dramatically reduced α diversity of Post-ABX Stool samples (Figure 3B). Finally, recipients’ cecal contents harvested after FMT at study endpoint displayed regained α diversity metrics, indicating the successful engraftment of donor microbes (Figure 3B). Taken together, these results demonstrate the success of our FMT design (32).

To examine effects of FMT and ICB on Obese versus VSG microbiome recipients, endpoint cecal contents in IgG2a controls compared to αPD-1 intervention were examined from mice examined in Figure 1 and Supplemental Table 1. Obese FMT compared with VSG FMT recipient contents revealed only moderate variation at the family level (Figure 3C). Interestingly, αPD-1 ICB therapy induced unique changes to the microbiome in Obese FMT recipients that were absent in VSG FMT αPD-1 recipients as well as IgG2a controls of either donor group (Figure 3C). In the Obese FMT αPD-1–treated mice, increases were observed in family *Chitinophagaceae*, family *Ruminococcaceae,* and genus *Oscillibacter* (Figure 3C and Supplemental Table 1). In VSG FMT αPD-1–treated mice, family *Peptostreptococcaceae*, a member of order Clostridiales, was uniquely elevated among the 4 groups (Figure 3, C and D). Order Clostridiales was also uniquely elevated in VSG FMT αPD-1–treated mice (Figure 3E), which significantly negatively associated with reduced tumor volume by microbiome multivariable associations in linear models (MaAsLin2; ref. 39) correlation analysis (Figure 3F). The gut microbiome, including members of order Clostridiales, produces or modifies metabolites, which prompted us to next conduct metabolomic analysis (40).

### Elevated circulating BCAA concentrations correlate with improved antitumor immunity.

**Figure 4:**
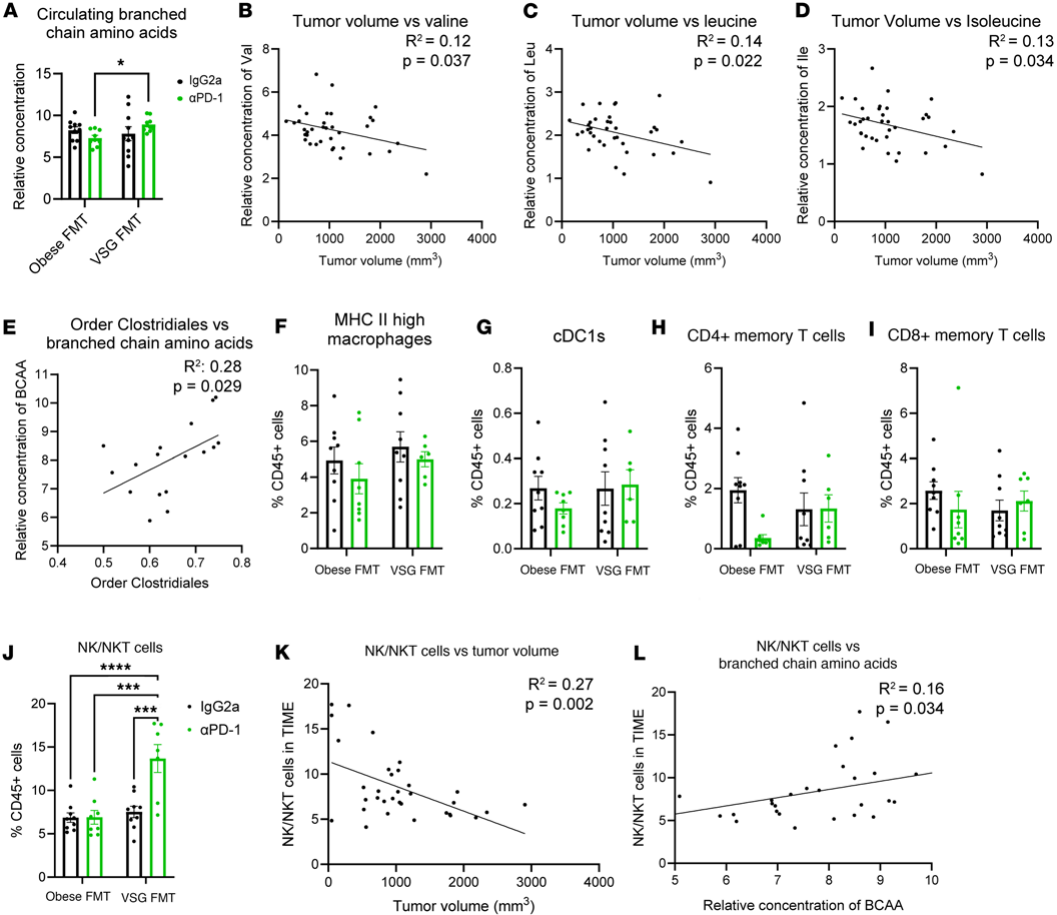
Elevated circulating branched chain amino acids were detected after FMT of VSG donor microbes, which correlated with improved antitumor immune response and NKT cells. (**A**) Relative concentration of circulating branched chain amino acids (BCAAs, sum of valine, leucine, and isoleucine) quantified by GC-MS analysis of plasma. Data are presented as mean ± SEM with 2-way ANOVA comparisons for *n* = 9–10 per group. Black bars represent groups treated with IgG2a isotype control, while green bars represent groups treated with α-PD1 ICB. (**B**–**D**) Circulating individual BCAAs correlated with tumor volume at endpoint via linear regression analysis for valine (**B**; *R*^2^ = 0.12, *P* = 0.037, *n* = 36), leucine (**C**; *R*^2^ = 0.14, *P* = 0.022, *n* = 36), and isoleucine (**D**; *R*^2^ = 0.13, *P* = 0.034, *n* = 36). (**E**) Abundance of order Clostridiales members in cecal contents at endpoint were correlated with relative concentration of circulating BCAAs via MaAsLin2 analysis. *R*^2^ = 0.28, *P* = 0.029, *n* = 17. (**F**–**J**) Flow cytometric analysis of the tumor immune microenvironment (TIME) is shown as M1-like MHC II^hi^ macrophages (**F**; CD11b^+^Ly6C^–^Ly6G^–^F480^+^MHC II^hi^), classical dendritic cells type 1 (**G**; cDC1s, CD11c^+^MHCII^+^CD11b^lo^CD10^hi^), CD4^+^ memory T cells (**H**; CD4^+^CD44^+^CD69^–^), CD8^+^ memory T cells (**I**; CD8^+^CD44^+^CD69^–^), or NK/NKT cells (**J**, NK1.1^+^) out of total CD45^+^ immune cells. Data are presented as mean ± SEM with 2-way ANOVA comparisons for *n* = 6–9 per group. *P < 0.05; ***P < 0.001; ****P < 0.0001. (**K**) Percentage of NK/NKT cells out of total immune cells within the TIME were correlated with tumor volume at endpoint via linear regression analysis. *R*^2^ = 0.27, *P* = 0.002, *n* = 33. (**L**) Percentage of NK/NK T cells out of total immune cells within the TIME were correlated with circulating BCAA levels via linear regression analysis. *R*^2^ = 0.16, *P* = 0.034, *n* = 29.

Order Clostridiales and other microbes alter circulating BCAAs, including leucine, isoleucine, and valine concentrations in the host (41). Because BCAAs are known to improve BC outcomes in preclinical models (42), we next determined the effects of VSG and ICB on BCAA concentrations and antitumor immunity. Metabolomic profiling by gas chromatography–mass spectrometry (GC-MS) of fasted plasma samples collected at study endpoint was conducted on microbially derived metabolites, with 67 metabolites reported in Supplemental Table 2. Compared with αPD-1–treated Obese FMT recipients, circulating BCAAs (the sum of leucine, isoleucine, and valine) were significantly greater in VSG FMT recipients after αPD-1 immunotherapy (Figure 4A). Linear regression analysis of individual BCAAs and tumor volume showed significantly negatively associated findings across all 3 BCAAs, with greater valine, leucine, or isoleucine concentrations in smaller tumors and reductions in BCAA concentrations in larger tumors (Figure 4, B–D). Order Clostridiales relative abundance was found to be significantly positively correlated with BCAAs by MaAsLin2 analysis (Figure 4E).

### NKT cells were uniquely upregulated in VSG FMT recipients after αPD-1 immunotherapy and correlated with elevated circulating BCAAs.

BCAAs regulate the adaptive immune system (43), which could impact antitumor immunity. Flow cytometry was conducted on the tumors excised at endpoint to investigate the tumor immune microenvironment (TIME) using separate innate and adaptive panels (gating schema in Supplemental Figure 6). Interestingly, despite great differences in tumor progression (Figure 1, B and C), there were no significant changes in tumor immune cell content overall (Supplemental Figure 7B). Nor were there significant differences among the groups across many specific immune cells, including M1-like MHCII^hi^ macrophage subtype, conventional dendritic cells (cDCs) overall, cDC1 or cDC2 subtypes, CD4^+^ T cells and memory subsets, or CD8^+^ T cells and memory subsets between Obese and VSG FMT or ICB recipients (Figure 4, F–I, and Supplemental Figure 7). Surprisingly, NK and NKT cells, identified by the NK1.1 marker, were impacted in a manner that eclipsed other immune cell differences. NK1.1^+^ NK/NKT cells were significantly and uniquely upregulated in VSG FMT recipients after αPD-1 immunotherapy (Figure 4J), with an almost 2-fold increase in ICB-treated mice compared with IgG2a controls detected only in the mice after receiving microbes from mice that had bariatric surgery. NK/NKT cells are effective in antitumor immunity (44–46); therefore, it follows that NK/NKT cell content significantly negatively correlated with endpoint tumor volume by linear regression analysis; the lower the NK/NKT cell content, the larger the tumors observed (Figure 4K). When immune cells were examined by linear regression analysis for correlations between circulating microbially derived metabolite concentrations and immune cell content within the TIME, BCAA concentrations significantly positively correlated with NK/NKT cell TIME content, with 64 other metabolites not significantly correlated (Figure 4L). There were no significant changes in circulating BCAA levels between Obese and VSG donor mice (Supplemental Figure 8A). However, in donors from which the cecal content was isolated for FMT in Figure 1, NKT cell enrichment was significantly elevated in tumors from VSG donors when compared with Obese donors (Supplemental Figure 8B). Importantly, NK cells were not enriched (data not shown). Lastly, in the same donor mice, BCAA concentration correlated positively and significantly with NKT cell enrichment (Supplemental Figure 8C). Immunometabolic analysis of both donors and FMT recipients suggests a critical contribution of BCAAs and NK/NKT cells to improved response to immunotherapy in mice after VSG and, importantly, those receiving fecal content of donors after VSG, which prompted us to investigate BCAA supplementation to determine direct impacts on response to ICB.

### BCAA supplementation mimics the effects of the post–bariatric surgery microbiome to boost immunotherapy effectiveness through elevations in invariant NKT cells.

**Figure 5:**
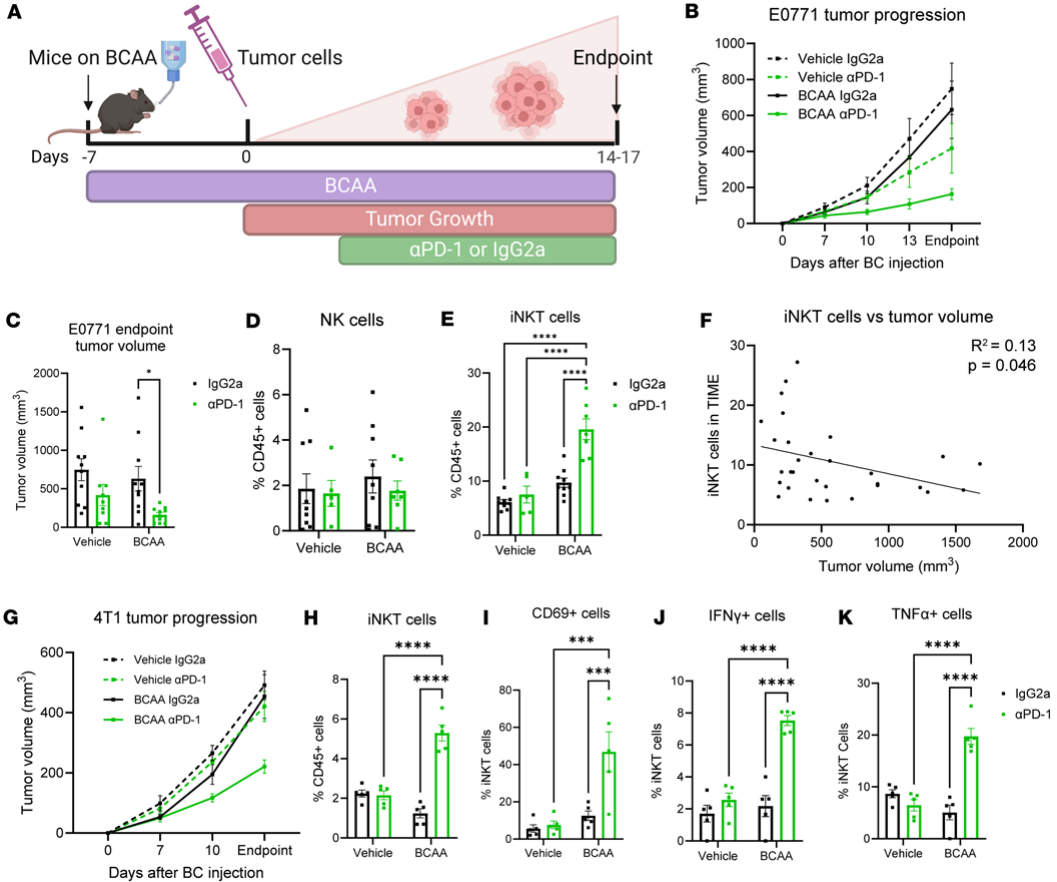
Branched chain amino acid supplementation mimics the effects of the post–bariatric surgery microbiome. (**A**) Study schema: Female C57BL/6J mice were purchased from The Jackson Laboratory at 4 weeks of age. On day –7, a branched chain amino acid (BCAA) cocktail (15 g/L leucine, 15 g/L isoleucine, and 15 g/L valine) was administered via drinking water for the duration of the study. Following 7 days of BCAA supplementation, E0771 BC cells were injected into the fourth right mammary fat pad and monitored for 3 weeks. αPD-1 ICB therapy or IgG2a isotype control was administered at 200 μg/mouse intraperitoneally every 3 days from tumor injection to endpoint. (**B**) E0771 tumor progression was recorded by digital caliper. *n* = 9–10 per group. (**C**) E0771 tumor volume at endpoint is presented as mean ± SEM with comparisons determined by 2-way ANOVA; *n* = 9–10 per group. (**D** and **E**) Flow cytometric analysis of E0771 tumor immune microenvironment (TIME) shown as NK cells out of total CD45^+^ immune cells (**D**, NK1.1^+^CD3^–^) and invariant NKT (iNKT) cells (**E**; CD3^+^, PBS57-loaded mouse CD1d tetramer^+^) out of total CD3^+^ cells. Mean ± SEM with 2-way ANOVA for *n* = 5–9 per group. (**F**) Percentage of iNKT cells out of total CD3^+^ cells within E0771 TIME were correlated with tumor volume at endpoint via linear regression analysis. *R*^2^ = 0.13, *P* = 0.046, *n* = 30. (**G**) 4T1 tumor progression in BALB/c mice was recorded by digital caliper. *n* = 5 per group. (**H**–**K**) Flow cytometric analysis of 4T1 TIME shown as iNKT cells (**H**, PBS57-loaded mouse CD1d tetramer^+^) out of total CD45^+^ cells, and CD69^+^ iNKT cells (**I**), IFN-γ^+^ iNKT cells (**J**), and TNF-α^+^ iNKT cells (**K**) out of total iNKT cells. Data presented as mean ± SEM with 2-way ANOVA for *n* = 5 per group. *P < 0.05; ***P < 0.001; ****P < 0.0001.

To directly test the relationship between circulating BCAA concentrations, NK/NKT cell content, and response to immunotherapy, 2 strains of mice were treated with oral BCAA supplementation to examine 2 different BC models. Female C57BL/6J or BALB/cJ mice were placed on LFD as in Figure 1 and supplemented with autoclaved water containing equal concentrations of valine, leucine, and isoleucine or vehicle controls (Figure 5A) as previously published (43). E0771 and 4T1 orthotopic injections and immunotherapy, respectively, were performed as described in Figure 1A. As in FMT studies, water intake was monitored, and BCAA supplementation did not affect water intake, body weights, or mammary adipose weight compared to controls (Supplemental Figure 9, A–C, and Supplemental Figure 11, A and B). Measurement of tumor progression demonstrated that mice receiving BCAA supplementation displayed no change in tumor progression; however, in combination with ICB, both models demonstrated significantly improved responses to αPD-1 (Figure 5, B, C, and G, Supplemental Figure 9, D–G, and Supplemental Figure 11, C–F). Mice receiving vehicle displayed a moderate 44.1% reduction in tumor progression after αPD-1, while mice receiving BCAA supplementation showed a significant 74.2% reduction in tumor volume after ICB in the E0771 model (Figure 5, B and C). Interestingly, the 4T1 model showed no response to ICB in the vehicle group, demonstrating resistance to immunotherapy. However, a 51% reduction in tumor progression after ICB in the BCAA-supplemented group was evident in the 4T1 model (Figure 5G). Similar to FMT studies, BCAA supplementation did not cause significant changes to the TIME in many innate or adaptive immune cell types, as measured by flow cytometry (Figure 5D and Supplemental Figure 9, H–K, gating scheme in Supplemental Figure 10). Because differences between NKT but not NK cells were evident in donor tumors with and without bariatric surgery, a tetrameric α-galactosylceramide analog, PBS57, was used to specifically stain for the iNKT cell subtype (gating scheme in Supplemental Figure 12) and separate NK and NKT cells present in the TIME. NK cell content was unchanged by BCAA supplementation (Figure 5D); however, iNKT cell content was uniquely increased by 2- to 4-fold in mice receiving BCAA supplementation in combination with αPD-1 ICB in both the E0771 model (Figure 5E) and the 4T1 model (Figure 5H). iNKT cell TIME content correlated significantly negatively with tumor volume in both models (Figure 5F and Supplemental Figure 11H). Thus, BCAA supplementation mimicked the effects of the post–bariatric surgery microbiome, with a combination therapy of BCAAs plus immunotherapy demonstrating potent antitumor effects on BC in 2 preclinical models.

Importantly, BCAA supplementation not only resulted in increased iNKT cell content within the TIME but also elevated iNKT cell activation markers and proinflammatory cytokine production. CD69 expression was quantified as a marker of early iNKT cell activation (47). BCAA supplementation in combination with αPD-1 ICB resulted in increased activation marker CD69^+^ iNKT cell staining, which was 3.7-fold greater than IgG2a control therapy in the BCAA-treated mice but not the vehicle controls (Figure 5I).

Flow cytometric analysis of the TIME from Donor B FMT (Figure 2) demonstrated increased iNKT cell content as well as elevated activation marker CD69^+^ iNKT cell content in the tumors of mice receiving postsurgery FMT in combination with immunotherapy (Supplemental Figure 14, A and B, gating scheme in Supplemental Figure 13). iNKT cell content in the TIME significantly negatively correlated with tumor volume (Supplemental Figure 14C). No additional significant changes in other innate or adaptive immune cell types in the TIME were observed (Supplemental Figure 14, D–G).

To determine the specific subtype of iNKT cells present in response to BCAA supplementation, intracellular flow cytometry staining revealed elevated content of both IFN-γ and tumor necrosis factor α (TNF-α) (Figure 5, J and K) with BCAA treatment compared with controls. Secretion of these proinflammatory cytokines classifies the iNKT cells present in the TIME as belonging to the antitumor iNKT1 subtype (48). Taken together, our results show that oral BCAA supplementation replicated the effects of an FMT of post–bariatric surgery gut microbes on immune reprogramming to a more potent antitumor TIME.

### BCAA treatment increased iNKT cell production of proinflammatory cytokines in vitro.

**Figure 6:**
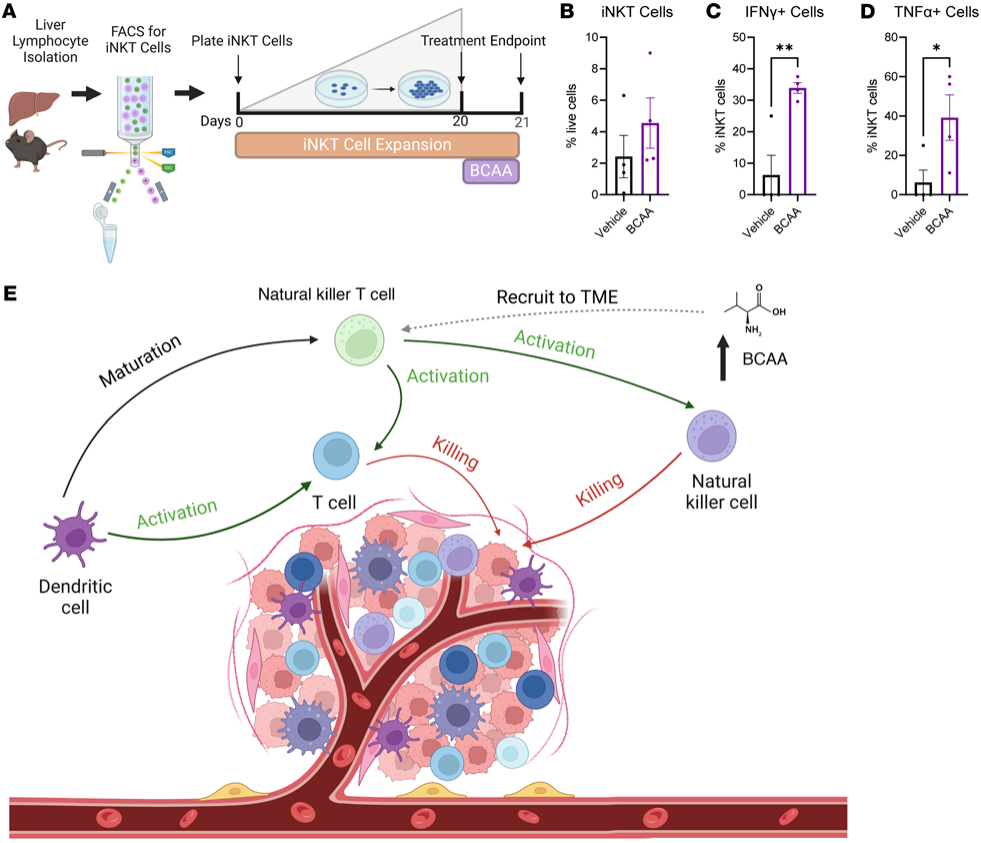
Branched chain amino acid treatment increases iNKT cell production of proinflammatory cytokines. (**A**) Study schema: Liver lymphocytes were isolated from female C57BL/6J mice and FACS was used to isolate iNKT cells via PBS57-loaded mouse tetramer staining. Cells were expanded in culture for 20 days. On day 20, cells were treated with a branched chain amino acid (BCAA) cocktail (10 mM leucine, 10 mM isoleucine, and 10 mM valine) for 24 hours. On day 21, cells were stained for flow cytometric analysis. (**B**–**D**) Flow cytometric analysis of cultured cells at endpoint shown as iNKT cells (**B**, PBS57-loaded mouse tetramer^+^) out of total live cells, IFN-γ^+^ cells (**C**) and TNF-α^+^ cells (**D**) out of iNKT cells. Data presented as mean ± SEM with 2-tailed Student’s *t* test for *n* = 4 wells per group. *P < 0.05, **P < 0.01. (**E**) Proposed model: NKT cells are induced to mature by dendritic cells. Mature NKT cells activate antitumor immune cells including T cells and NK cells, which induce cancer cell death. Findings presented suggest that BCAAs are elevated in mice after FMT from VSG donors and are essential drivers of the NKT cell antitumor immune response. Solid lines indicate known interactions, while dashed lines represent proposed interactions.

Lastly, to investigate the direct activation of iNKT cells by BCAAs, iNKT cells were treated with BCAAs in vitro. Primary iNKT cells were isolated from female C57BL/6J mouse livers via fluorescence-activated cell sorting (FACS) (Figure 6A). Ex vivo*,* cells were expanded in culture for 20 days. On day 20, expanded iNKT cells were treated with BCAAs for 24 hours following previously published study designs (43) prior to endpoint analysis by flow cytometry. BCAA treatment minimally but not significantly elevated iNKT cell proliferation (Figure 6B). Within iNKT cells, greatly increased abundance of IFN-γ^+^ and TNF-α^+^ cells were detected among BCAA-treated cells relative to controls, which essentially expressed no cytokines in 3 of the 4 replicates (Figure 6, C and D). In summary, these findings indicate that BCAAs play a direct role in iNKT cell cytokine production, which may underlie the potent antitumor immune response in mice supplemented with BCAAs or those after bariatric surgery FMT.

## Discussion

A history of bariatric surgery reduces cancer risk for cancers associated with obesity as well as obesity-independent cancers and improves the outcomes of a subsequent cancer diagnosis (3). Our group has previously tested the impact of bariatric surgery on cancer outcomes in a preclinical model demonstrating improved response to ICB following VSG-induced weight loss relative to obese controls receiving a sham surgery (16). However, the mechanisms behind the improved ICB efficacy after bariatric surgery were unknown. A potential mediator of reduced risk and improved outcomes is changes to the gut microbiome, which is substantially and durably modified following bariatric surgery (19–21), resulting in a unique post–bariatric surgery phenotype. Thus, this study investigated whether potential changes to the microbiome after bariatric surgery can mediate the improved response to immunotherapy in BC. This report demonstrates that FMTs are sufficient to increase immunotherapy effectiveness, meaning that the boost to antitumor immunity after bariatric surgery is transferrable from donor to recipient through the gut microbiome. Recipients treated with microbes from VSG donors displayed a significantly improved response to αPD-1 ICB when compared with mice treated with microbes from obese donors or control mice with no FMT, resulting in both delayed tumor progression and blunted tumor burden at endpoint. Experimental control mice lacking both ABX and FMT mirrored the response to immunotherapy in our Obese FMT group, indicating that the improved response is due to the VSG FMT and not inhibition of response caused by Obese FMT. Remarkably, stool samples collected from obese patients before surgery or those same patients’ stool after bariatric surgery demonstrated delayed tumor progression after transplantation of the weight-loss fecal microbiome. Thus, even in the absence of ICB, patient fecal content after bariatric surgery demonstrated antitumor potential that was not observed using murine samples after VSG, potentially due to differences between human and mouse diets or other factors undefined at this time that are known to play a large role in microbiome composition (32). Excitingly, we observed improved responses to αPD-1 ICB in our human-to-mouse FMT models. Last, a striking increase in NKT cells in tumors from donor mice after bariatric surgery and tumors in recipient mice after FMT of cecal contents from bariatric surgery donors suggested an underlying pathway driving increased NKT cell antitumor immunity. We demonstrated that circulating BCAAs positively correlated with NKT cells across multiple studies. Indeed, we tested the hypothesis that BCAAs may be mediating VSG-dependent benefits and demonstrated that with BCAA supplementation, iNKT cells were elevated in tumors and tumor burden was significantly reduced compared with nonsupplemented controls. Together, these findings suggest a role for bariatric surgery–induced modification of microbially derived metabolism of BCAAs that amplify iNKT cell antitumor immunity.

Transcriptomic analysis of tumors revealed a much more robust antitumor immune response in the recipients of VSG FMT compared with those receiving Obese FMT. Proinflammatory and antitumor pathways were upregulated by αPD-1 in the VSG recipients, pathways that were not upregulated in the Obese FMT recipients following αPD-1 ICB treatment. The combination of VSG microbes and αPD-1 ICB resulted in elevation of specific pathways such as HALLMARK_IL2_STAT5_signaling and HALLMARK_Allograft_Rejection, which are indicators of a strong immune response in these mice that had the smallest tumors.

The blunted tumor progression, despite recipient mice demonstrating no differences in body weights and metabolic or other parameters, emphasize a distinctly gut-microbiome-derived impact on the TIME. Previous work identified either specific microbes or certain microbially derived metabolites that mediate an improved response to ICB, including *Bacteroides* spp., *Bifidobacterium* spp., and *Lactobacillus* spp., as well as metabolites such as indole derivatives and SCFAs (28, 49–53). Bariatric surgery is known to induce durable changes to the composition of the gut microbiome in patients (21). Therefore, we examined the microbial composition of donor and recipient cecal contents to provide insight into potential mediators of antitumor immunity after bariatric surgery. Cecal content was first examined from donors that either received sham control surgery or those treated with VSG that lost 28% of body weight (as previously reported; ref. 16). Interestingly, family *Peptostreptococcaceae* was moderately increased in the cecal contents of VSG donors compared with Obese donors. Importantly, the same family, *Peptostreptococcaceae*, was also uniquely elevated in the cecal contents of VSG FMT recipients that received αPD-1 ICB. Of note, this family of microbes, a member of order Clostridiales, has previously been associated with elevated colonic BCAAs in preclinical models (54). Indeed, targeted metabolomics on plasma samples collected at endpoint from both donor and recipient mice identified elevated circulating BCAA levels in VSG FMT recipients treated with αPD-1 ICB out of dozens of metabolites quantified, as well as a positive correlation between abundance of order Clostridiales in the cecum and circulating BCAA levels.

BCAAs impact T cell differentiation and improve immune function (43, 55); therefore, immune changes after FMT were examined in the TIME. Surprisingly, across multiple studies with varying tumor sizes, there were no changes in myeloid cell content, including monocyte-derived suppressor cells and macrophages, DCs, or T cells, including memory subsets. The single cell type dramatically altered across multiple studies presented herein were NKT cells, which were increased in the donor TIME after VSG and in recipient tumors after FMT of microbes from VSG donors and αPD-1 ICB treatment. NKT cells are a subset of T cells that respond to lipid antigens and are known to be modulated by the microbiome (56, 57). These multifunctional cells primarily serve as architects of the antitumor immune response (Figure 6E). In response to external stimuli, NKT cells release cytokines that drive immune activation or immunosuppression (58, 59), and NKT cell function is critical for αPD-1 ICB effectiveness, potentially through reinvigorating exhausted CD8^+^ T cells to drive sustained antitumor immune responses (60, 61). We found that NK/NKT cell content was increased in mice with the smallest tumors resulting from αPD-1 treatment, but only in the VSG FMT group. Most interestingly, NK/NKT cell content within the TIME positively correlated with circulating BCAA concentrations in VSG FMT recipients, while NKT cells specifically but not NK cells correlated with circulating BCAA concentrations in VSG FMT donors and postsurgery FMT recipients. This correlation suggested a potential role for BCAAs in modulating NKT cell antitumor responses after αPD-1 ICB, which we next evaluated as a neoadjuvant therapy to boost ICB effectiveness.

Lastly, to test the direct interaction between elevated BCAA levels and NK/NKT cell content in the TIME, mice were supplemented with BCAAs in drinking water. Dietary supplementation of BCAAs has previously been associated with reduced risk or metastasis of certain cancer types, including colorectal cancer and BC, but the mechanisms were not defined in detail (42, 62–64). NKT cells can be divided into subsets based on the structure of their TCR α-chain (invariant type 1 and diverse type II) and their cytokine production profile (Th1-like iNKT1, Th2-like iNKT2, Treg-like, and more) (48, 65). iNKT1 cells are classically immune activating, secreting cytokines such as IFN-γ and TNF-α, while iNKT2 cells are considered immunosuppressive, secreting cytokines such as IL-4 and IL-13 (48). To confirm the subtype of NKT cells present in the TIME of BCAA-supplemented mice, a tetrameric α-galactosylceramide analog, PBS57, was used to specifically stain for iNKT cells (66). BCAA supplementation potently improved αPD-1 ICB effectiveness, with significant blunting of tumor progression and reduction in endpoint tumor burden. The improved tumor outcomes were accompanied by increased iNKT cell content within the TIME uniquely in BCAA supplementation recipients treated with αPD-1 ICB. NK cell content remained unchanged by BCAA supplementation and ICB treatment. Tetramer-positive iNKT cells correlated negatively with tumor volume at endpoint and demonstrated increased intracellular IFN-γ and TNF-α, indicating an improved antitumor immune response after BCAA supplementation. Furthermore, ex vivo BCAA treatment of iNKT cells demonstrated a direct impact on elevated antitumor cytokines, with activation markers and cytokines increased in vitro similarly to those observed in vivo with BCAA supplementation studies.

In sum, the work of our group and others suggested that bariatric surgery was protective to reduce tumor burden, but the underlying mechanisms were unresolved. Work presented herein demonstrates that FMT of microbes from mice with previous VSG into naive lean recipient mice was sufficient to reproduce the improved response to immunotherapy observed in FMT donor mice. These findings were further supported by FMTs of human stool samples collected prior to and after VSG-induced weight loss, which resulted in tumor regression alone and when combined with αPD-1 ICB treatment. Circulating BCAAs were uniquely increased in VSG FMT αPD-1 mice, correlating with NKT cell content within the TIME. Importantly, BCAA supplementation was sufficient to induce an improved response to immunotherapy via increased NKT cell content within the tumor in 2 complementary BC models, one of which was highly ICB resistant. There are several limitations to this work. Our study examines the impact of changes to the gut microbiome following bariatric surgery on BC progression and treatment response; however, tumor- or tissue-specific microbes may also impact outcomes separate from or in addition to gut microbes. The FMTs were conducted using both mouse and human transplants into 1 murine model of BC, the C57BL/6J strain with orthotopic injection of E0771 BC cells, which should be tested in additional models, including genetically engineered mouse models (GEMMs) that spontaneously develop cancer (e.g. Pymt or C3(1)-Tantigen models). However, the BCAA supplementation studies were demonstrated in 2 mouse strains using 2 syngeneic BC cell lines, the C57BL/6J/E0771 model and the BALB/cJ/4T1 model. These data suggest an interaction between microbially derived BCAAs and NKT cells that may be manipulated to improve ICB response rates. Less than 25% of triple-negative BC patients currently respond to ICB therapy (67). Thus, this study is a crucial step in understanding immunotherapy response and developing strategies to improve ICB efficacy for patients.

## Methods

### Sex as a biological variable.

Sex was considered as a biological variable. Male BC accounts for less than 1% of reported BC cases worldwide (68); hence, female mice were used in all experiments.

### Animals, diet, and ABX exposure.

Four-week-old female specific pathogen–free C57BL/6J mice (stock number 000664) were purchased from The Jackson Laboratory. Diet and housing details are available in the Supplemental Methods. After a 2-week acclimation period, mice were treated with an ABX cocktail (0.5 g/L vancomycin, 1 g/L neomycin, 1 g/L ampicillin, and 1 g/L cefoperazone in autoclaved water) or plain autoclaved water in water bottles for 8 days to ablate the commensal gut microbiome. Water intake was measured by water bottle weight daily. Body weight was measured weekly until the study endpoint.

### FMT.

Fecal microbiota for FMT were collected from donors from our previously published study by Sipe et al. (16). Additional details are available in the Supplemental Methods.

To study the impact of transplanted microbiota on body weight, tumor progression, and response to immunotherapy, following ABX treatment, mice were assigned to 1 of 5 groups for the FMT controls or experimental groups (*n* = 10 mice per group). ABX cocktail was stopped 6 hours before the first gavage occurred and mice were given plain, sterile water in autoclaved water bottles. Mice were gavaged with 50 μL fecal slurry every 3 days for 2 weeks for a total of 4 FMT or control gavages as diagramed in the study design (Figure 1A), with E0771 cells injected after the second FMT gavage. Additional details are available in the Supplemental Methods.

### Human fecal collection and FMT studies.

Fecal samples were collected from 65-year-old (Donor A) and 40-year-old (Donor B) African American female patients before bariatric surgery (presurgery) and 6 months after bariatric surgery (postsurgery). Four-week-old specific pathogen–free female C57BL/6J mice (purchased from The Jackson Laboratory as described above) were placed on Mediterranean Diet (Research Diets Inc.) 24 hours after arriving at our facility. Mice were rested for 2 weeks and subjected to the same ABX and FMT study design as above for the mouse-to-mouse FMT experiment (Figure 1A). Additional details are available in the Supplemental Methods.

### BCAA supplementation study.

Mice were subjected to BCAA supplementation to attempt to mimic the effects of the post–bariatric surgery microbiome (Figure 5A). Five-week-old female specific pathogen–free C57BL/6J mice (The Jackson Laboratory) or BALB/cJ mice (The Jackson Laboratory, stock number 000651) were placed on LFD 24 hours after arriving at our facility. Mice were rested for 2 weeks prior to intervention and bedding was mixed between cages weekly to normalize baseline microbiomes across cages to reduce cage effects. After a 2-week acclimation period, mice were treated with a BCAA cocktail (15 g/L leucine, 15 g/L isoleucine, and 15 g/L valine in autoclaved water), while vehicle controls received plain autoclaved water in water bottles for the duration of the study, based on previous work by Yao et al. (43). After 8 days of supplementation, mice were injected with E0771 cells as above or 4T1 cells. ICB or IgG2a isotype control was administered as described in Supplemental Methods. Water intake was measured by water bottle weight daily. Body weight was measured daily until the study endpoint. Upon endpoint, tissues were collected as described in the Supplemental Methods.

### Cell culture and tumor cell implantation.

The E0771 BC cell line expressing luciferase, a gift from Hasan Korkaya (Wayne State University, Detroit, Michigan, USA), was cultured as previously described (16, 36, 37). 4T1 cells were purchased from ATCC (CRL-2539). Additional details are available in the Supplemental Methods.

### RNA isolation and sequencing.

Total RNA was extracted from tumors using the RNeasy Mini Kit (Qiagen, 74104) according to the manufacturer’s instructions. The integrity of RNA was assessed using an Agilent TapeStation and samples with RIN greater than 6.0 were used. mRNA-seq libraries for the Illumina platform were generated and sequenced at Azenta using the Illumina HiSeq 2 × 150 bp configuration following the manufacturer’s protocol. Analysis is described in the Supplemental Methods.

The ComplexHeatmap R package (69) was used to represent normalized and scaled gene expression values in heatmaps, where rows (genes) were clustered via complete linkage methods and *k*-means (*k* = 3) clustering while columns (samples) were split by groups. For volcano plots, DEGs with adjusted *P* values of 0.1 or less satisfied significance criteria consisting of an adjusted *P* value of less than 0.05 and log_2_|FC| of less than 0.58 using R version 4.3.1. RNA-seq results are available in the NCBI Gene Expression Omnibus (GEO GSE291031).

### Microbiota analysis.

Cecal contents were collected from donors described in Sipe et al. (16) and recipients at endpoint, flash-frozen, and stored at −80°C for analysis. Fresh stool samples were collected prior to commencing the ABX cocktail to establish a baseline and upon completion of the ABX regimen to determine to what extent ABX ablated the gut microbiota. Stool samples were collected on ice and stored at –80°C for analysis.

For DNA isolation, previously developed methods were followed (70). Additional details are available in Supplemental Methods. Paired-end reads were processed in R studio version 4.3.2 using the DADA2 pipeline (71) to correct sequencing errors and to determine amplicon sequence variants (ASVs). ASVs were assigned taxonomy by comparison to Ribosomal Database Project release 18.0 (72). Differential abundance of individual taxa was assessed using Corncob (73), while α diversity was calculated using vegan (74). MaAsLin2 was used for correlation analyses (39). Microbiota analysis results are available in the NCBI Sequence Read Archive (SRA PRJNA1233226).

### Metabolomic analysis.

Plasma collected at endpoint was analyzed for composition of circulating microbially derived metabolites, including SCFAs, amino acids, and other metabolites. Compositions were determined at the University of Chicago Duchossois Family Institute Host-Microbe Metabolomics Facility. Metabolites were isolated via methanol extraction. SCFA and amino acid groups were analyzed using GC-MS on an Agilent 7890A GC/Agilent 5975C MS detector with negative chemical ionization mode. Normalized peak areas were calculated by dividing raw peak areas of targeted analytes by averaged raw peak areas of internal standards. Correlation analyses were conducted using MaAsLin2 (39) in R studio version 4.3.2 or linear regression analysis in GraphPad Prism version 10.

### Flow cytometric analysis of tumors.

A portion of the excised tumors (~100 mg) were minced using scissors in RPMI 1640 media containing an enzyme cocktail mix for mouse tumor dissociation (Miltenyi Biotec) following established protocols (16, 36). In all studies, following red blood cell lysis (BioLegend), viability was determined by staining with Ghost dye (Tonbo Biosciences, Inc.) followed by FcR blocking (Tonbo Biosciences, Inc.). Cells were stained with fluorescently labeled antibodies and fixed in Perm/Fix buffer (Tonbo Biosciences Inc.). Additional details are available in the Supplemental Methods. Stained cells were analyzed using a Bio-Rad ZE5 flow cytometer at the UTHSC Flow Cytometry and Cell Sorting Core. Data were analyzed using FlowJo version 10 software with FlowAI plug in cleanup (75). Gating schemata are shown for both myeloid- and T cell–specific flow panels (Supplemental Figure 5) and NKT cell–specific staining (Supplemental Figures 10, 12, 13, and 15).

For intracellular staining, single-cell suspensions were stimulated with Cell Activation Cocktail (BioLegend, 423303) for 3 hours to allow accumulation of intracellular cytokines (35). Following cell surface marker staining, cells were fixed and permeabilized with Flow Cytometry Perm Buffer (Tonbo Biosciences, Inc.) overnight. Intracellular staining of IFN-γ and TNF-α was completed the following day.

### Ex vivo BCAA assays.

iNKT cells were isolated and expanded in culture following published protocols (76, 77). Details are available in Supplemental Methods. Sorting was performed using a Cytek Aurora cell sorter in the UTHSC Flow Cytometry and Cell Sorting Core. Cells were cultured as in previous publications (76). Following expansion, cells were treated with BCAAs (10 mM leucine, 10 mM isoleucine, and 10 mM valine) for 24 hours (43). At endpoint, intracellular flow cytometry staining was conducted as described above.

### Statistics.

All analyses and figure generation were performed in R studio version 4.3.2 or Prism version 10 (GraphPad Software, Inc.). Statistical differences between experimental groups were determined using 1-way or 2-way ANOVA (as noted in figure legends) with Fisher’s LSD test for individual comparisons. Outliers were identified and excluded based on the ROUT method with *Q* = 1% in GraphPad Prism. For body weight, water intake, and tumor volume over time within animals, data were treated as repeated measures. All data are shown as mean ± SEM. A *P* value of less than 0.05 was considered statistically significant. Sample size was determined by power analysis calculations and pilot experiments.

### Study approval.

Murine studies were performed with approval and in accordance with the guidelines of the Institutional Animal Care and Use Committee (IACUC protocol 24.0504.0) at the UTHSC and in accordance with the NIH *Guide for the Care and Use of Laboratory Animals* (National Academies Press, 2011). Human sample collection was conducted in accordance with the Declaration of Helsinki and approved by the Institutional Review Board of the UTHSC (IRB no. 20-07606-XP, approved June 2, 2022 and IRB no. 22-08722-FB, approved February 27, 2025). Written informed consent was obtained from all patients.

### Data availability.

RNA-seq data were deposited in the NCBI GEO (GSE291031). 16S microbiome sequencing data were deposited in the NCBI SRA database (NCBI BioProject ID PRJNA1233226). Supporting analytic code is included in Supplemental Methods. Data used to generate the figures are included in the Supporting Data Values file accompanying this manuscript.

## Author contributions

MSB, SCJ, LMS, JFP, and LM conceived and designed research. MSB, SCJ, MK, CTL, TSM, NBW, UAT, and DD developed methodology. MSB, SCJ, LMS, MK, CTL, TSM, UAT, JHE, EWG, MEP, ZTM, BAH, LGM, BLS, JAM, and PPN performed experiments. MSB, MK, NBW, UAT, JHE, JFP, and LM analyzed data. MJD, BDP, MVDM, JFP, and LM provided resources. JFP and LM supervised the research. MSB and LM drafted the manuscript; all authors revised the manuscript and approved the final submission.

## Supplementary Material

Supplemental data

Supplemental tables 1-2

Supporting data values

## Version 1 04/24/2025

In-Press Preview

## Version 2 06/09/2025

Electronic publication
