# Supplementary material for: The gut microbiome enhances breast cancer immunotherapy following bariatric surgery
Source: JCI Insight. 2025 Apr 24;10(11):e187683. doi: 10.1172/jci.insight.187683 (PMC12220976; doi:10.1172/jci.insight.187683)
Supplement: Supplemental data [file jciinsight-10-187683-s171.pdf]

Supplemental Figure 1

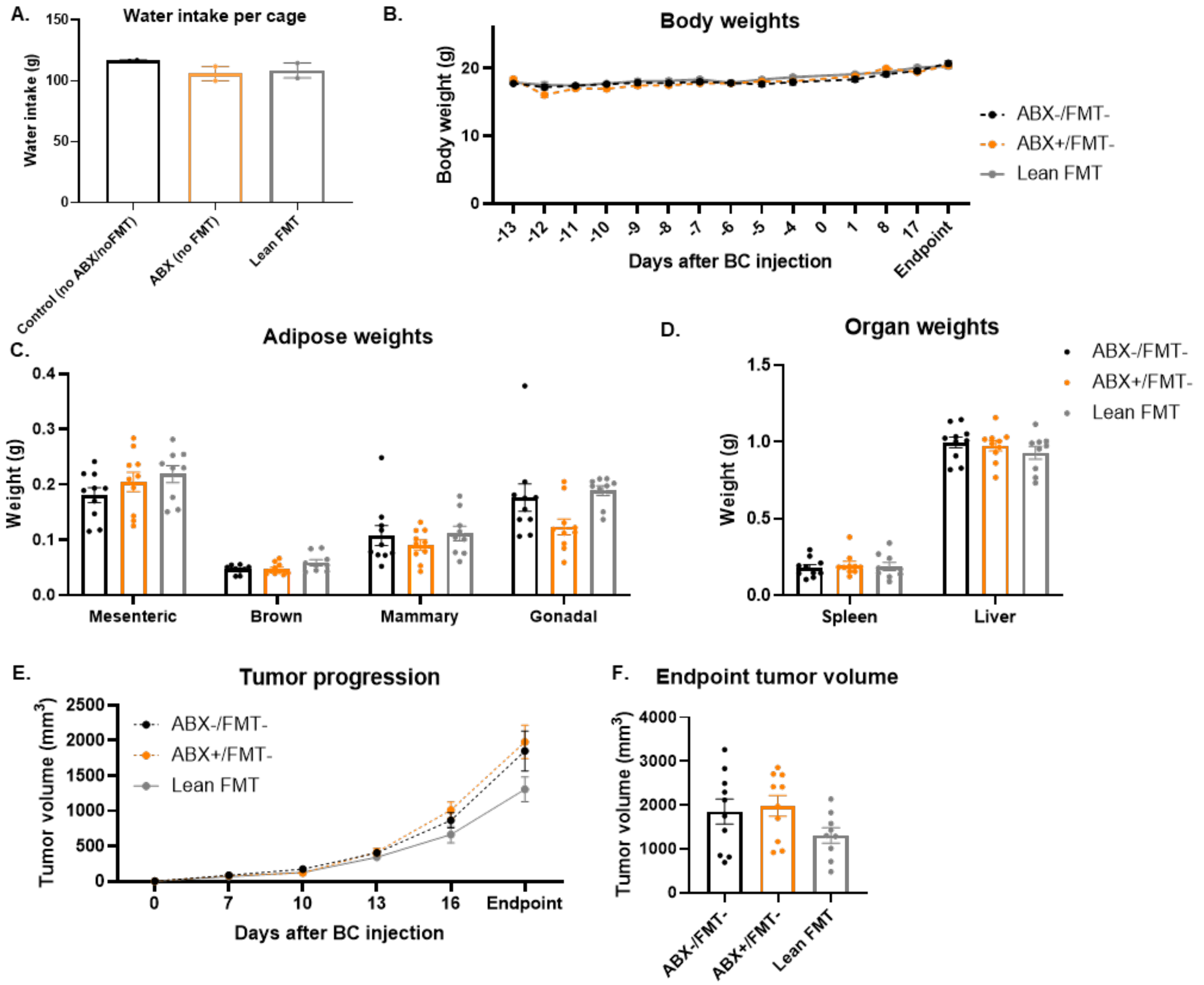

Supplemental Figure 1. Interventions did not impact body weight or fat mass of recipients.

(**A**) Antibiotic water intake was measured as mass in grams over the course of the entire 8-day antibiotic treatment intervention (Figure 1). Control cages received autoclaved water without antibiotics and the remaining 5 groups received antibiotic cocktail (controls not included in cartoon in Figure 1A). N = 2 cages per group with 4-5 mice per cage. (**B**) Body weights were measured for the duration of the study. Significance was determined by two-way ANOVA mixed effects model with Fisher’s LSD test; N = 9-10 per group. (**C**) Adipose tissue weights were measured as mass in grams for mesenteric, brown, mammary, and gonadal depots presented as mean ± SEM with comparisons determined by two-way ANOVA. N = 9 per group. (**D**) Organ weights were measured as mass in grams for spleen and liver. Data are presented as mean ± SEM with comparisons determined by two-way ANOVA. N = 9 per group. (**E**) Tumor progression was recorded by digital caliper with control mice (ABX-/FMT-) in the dashed black line, antibiotics only control in the dashed orange line (ABX+/FMT-), and mice receiving microbes through FMT from lean donors in the solid gray line.

Significance was determined by two-way ANOVA mixed effect model with Fisher's LSD test; N = 9-10 per group. (**F**) Tumor volume at endpoint presented as mean ± SEM with comparisons determined by one-way ANOVA. N = 10 per group. *$p<0.05$.

Supplemental Figure 2

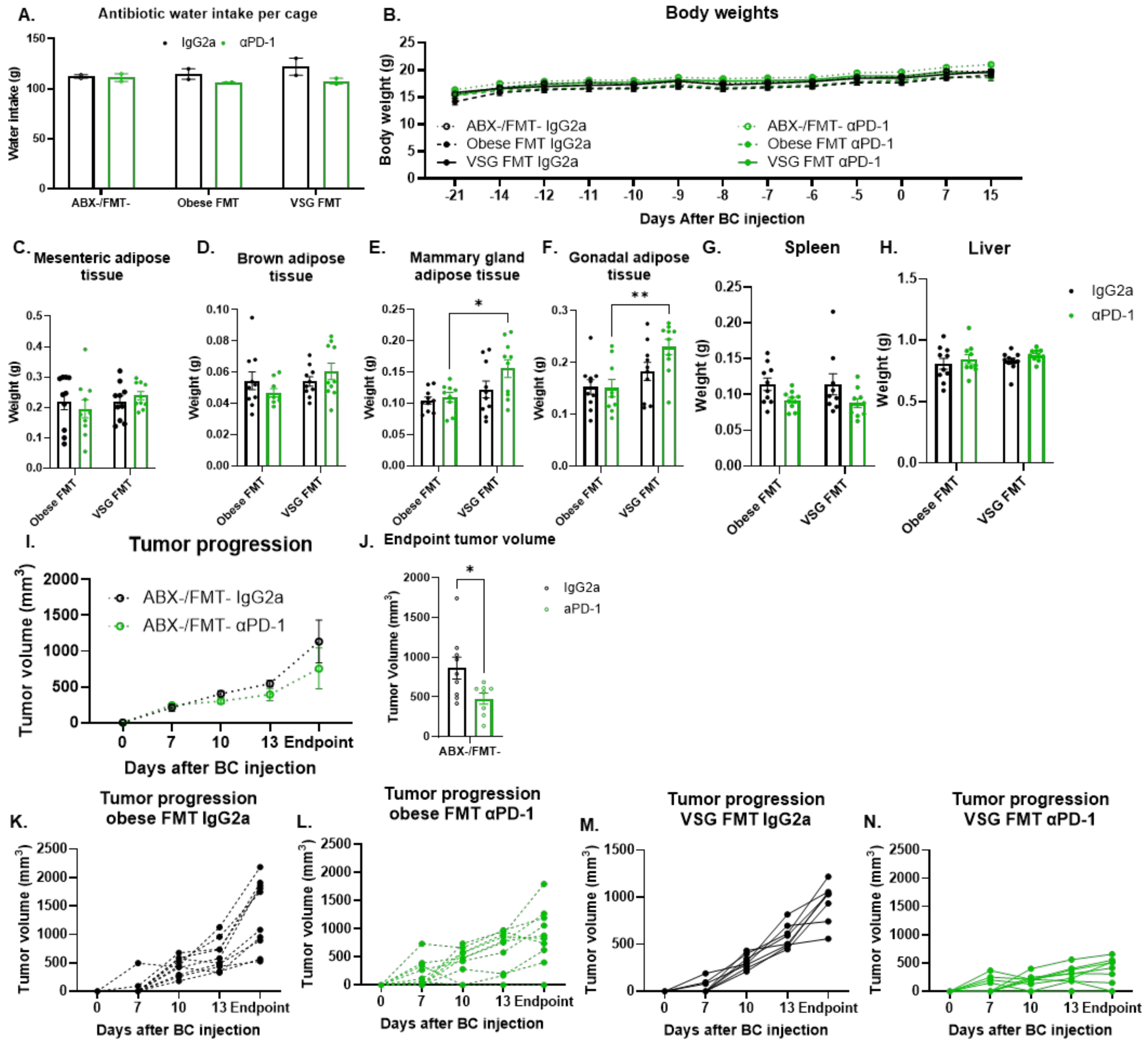

Supplemental Figure 2. Microbes transplanted from VSG donors improved ICB efficacy without impacting body weight or fat mass of recipients.

(**A**) Antibiotic water intake was measured as mass in grams at the cage level over the course of the entire 8-day antibiotic treatment. Control cages received autoclaved water without antibiotics. N = 2 cages per group. (**B**) Body weights were measured for the duration of the study. Significance was determined by two-way ANOVA with Fisher's LSD test; N = 10 per group. (**C-F**) Adipose tissue weights were measured as mass in grams for mesenteric (**C**), brown (**D**), contralateral uninjected mammary (**E**), and gonadal adipose (**F**) depots. Data are presented as mean ± SEM with comparisons determined by two-way ANOVA. N = 10 per group. (**G-H**) Organ weights were measured as mass in grams for spleen (**G**) and liver (**H**) presented as mean ± SEM

with comparisons determined by two-way ANOVA. N = 10 per group. (**I**) Tumor progression is shown over time with control mice (no antibiotic treatment or FMT gavage) that received αPD-1 ICB in the dashed green line and control mice that received IgG2a isotype control in the dashed black line. Significance was determined by two-way ANOVA mixed effect model with Fisher's LSD test; N = 10 per group. (**J**) Tumor volume at endpoint is presented as mean ± SEM with comparisons determined by Student's *t*-test. N = 10 per group. *p<0.05, **p<0.01. (**K-N**) Tumor progression in individual mice in each treatment group from Figure 1B, including obese FMT IgG2a (**K**), obese FMT αPD-1 (**L**), VSG FMT IgG2a (**M**), and VSG FMT αPD-1 (**N**); N = 8-10 per group.

Supplemental Figure 3

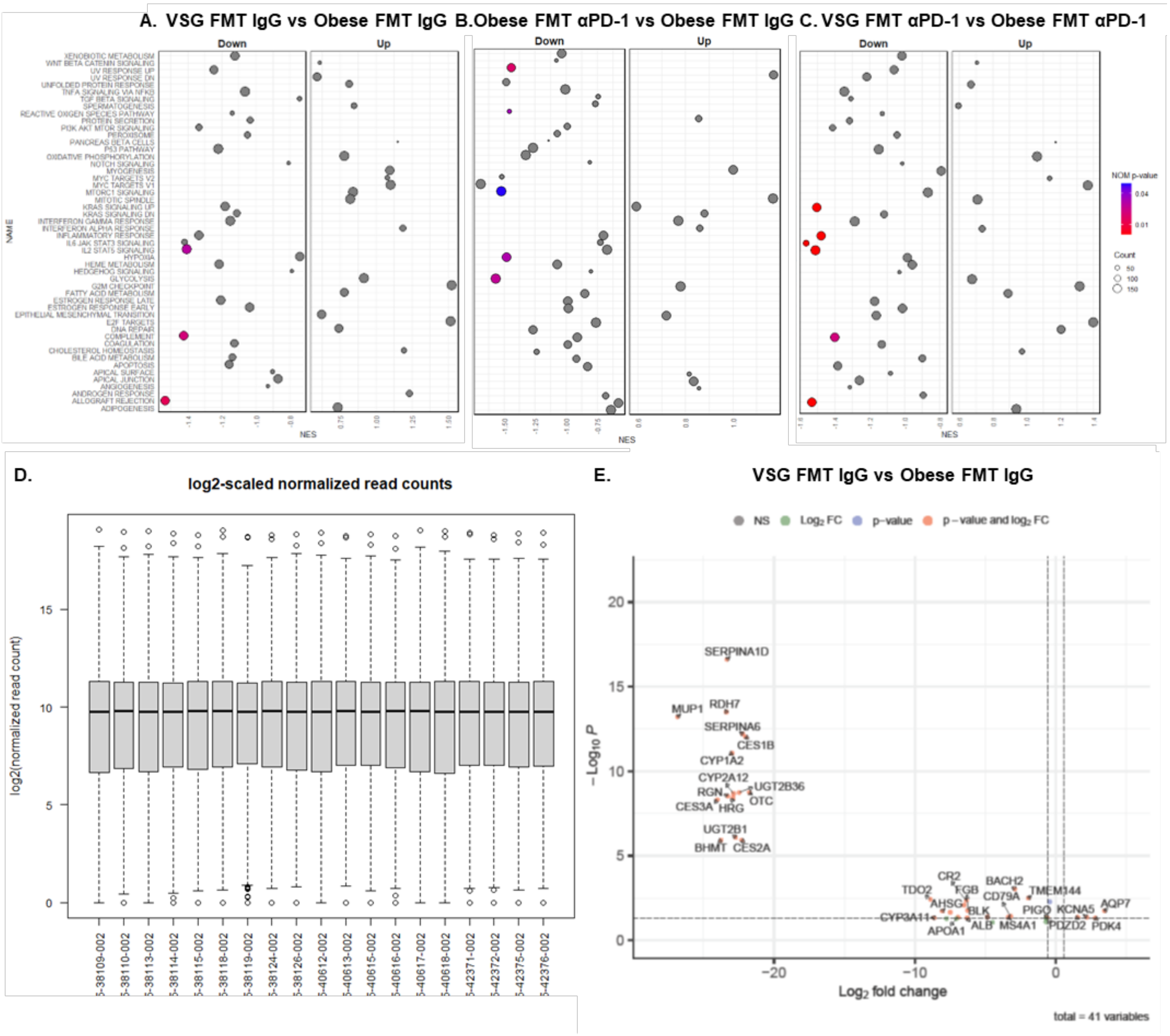

Supplemental Figure 3. Microbes transplanted from obese and VSG donors modulated immune-related HALLMARK pathways.

(**A-C**) Dot plots of GSEA results representing upregulated and downregulated HALLMARK pathways in FMT recipients plotted as VSG FMT IgG2a vs obese FMT IgG2a (**A**), obese FMT recipients plotted as αPD-1 ICB vs IgG2a isotype control (**B**), and FMT recipients plotted as VSG FMT αPD-1 ICB vs obese FMT αPD-1 ICB (**C**) with significant nominal p-values denoted by dot color and gene count signified by dot size. Pathways are ranked by normalized enrichment score (NES). (**D**) Log_2-scaled normalized read counts for all samples included in transcriptomic analysis. (**E**) Volcano plot of significant differentially expressed genes in tumors plotted as VSG FMT IgG2a vs obese FMT IgG2a with log_2 fold change (FC) of >1.5 and p-adjusted value <0.05. N = 3-6 per group.

Supplemental Figure 4

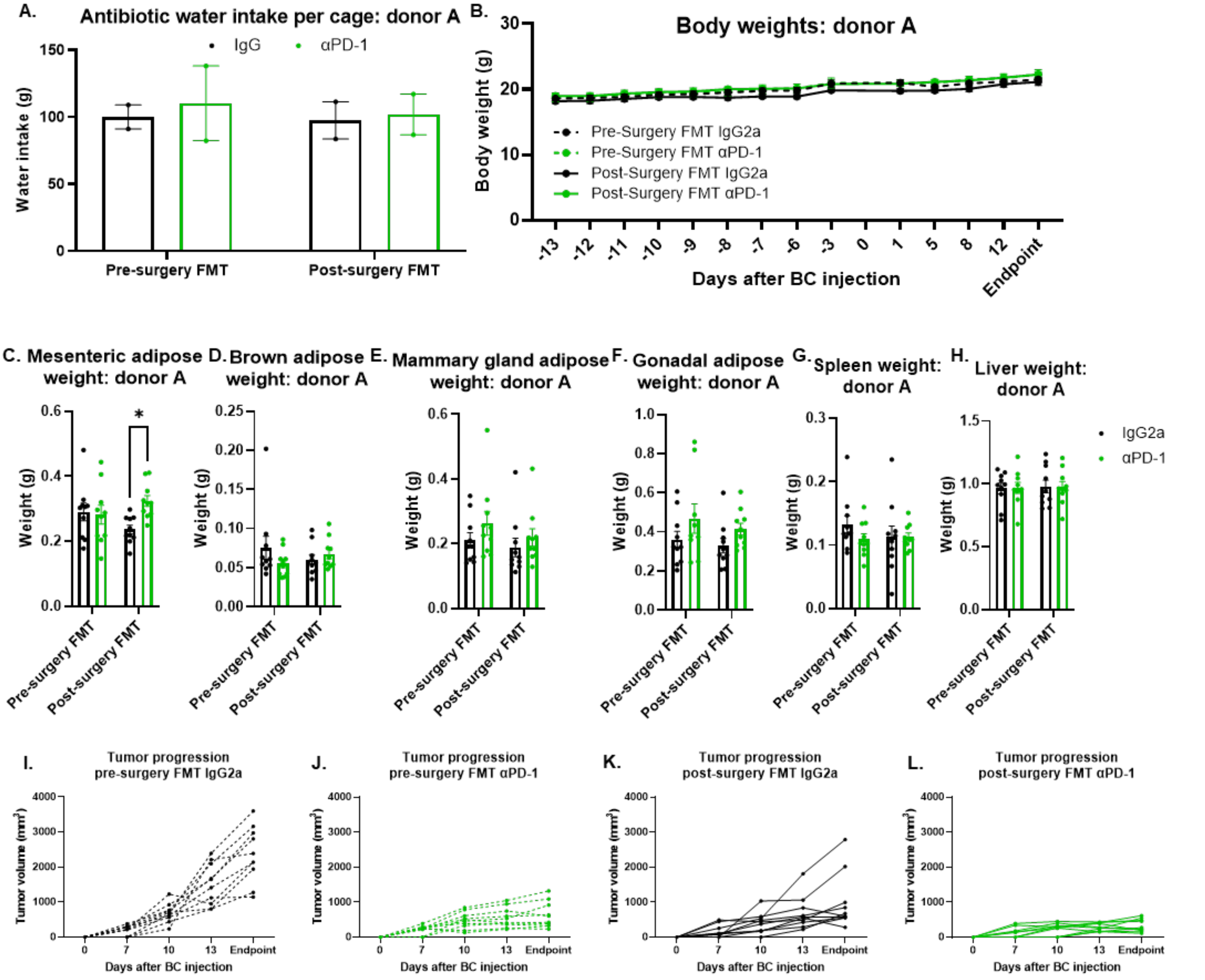

Supplemental Figure 4. Microbes from Donor A pre- and post-surgery do not impact body weight of recipients.

(**A**) Antibiotic water intake was measured as mass in grams at the cage level over the course of the entire 8-day antibiotic treatment. All cages received antibiotic cocktail. N = 2 cages per group, with N=5 mice per cage. (**B**) Body weights were measured over the duration of the study. Significance was determined by two-way ANOVA mixed effects model with Fisher's LSD test; N = 10 per group. (**C-F**) Adipose tissue weights were measured as mass in grams for mesenteric (**C**), brown (**D**), mammary (**E**), and gonadal adipose (**F**) depots. Data are presented as mean ± SEM with comparisons determined by two-way ANOVA. N = 10 per group. (**G-H**) Organ weights were measured as mass in grams for spleen (**G**) and liver (**H**). Data are presented as mean ± SEM with comparisons determined by two-way ANOVA. N = 10 per group. (**I-L**) Tumor progression in individual mice in each treatment group from Figure 2C, including pre-surgery FMT IgG2a (**I**), pre-surgery FMT αPD-1 (**J**), post-surgery FMT IgG2a (**K**), and post-surgery FMT αPD-1 (**L**); N = 10 per group. *$p<0.05$.

Supplemental Figure 5

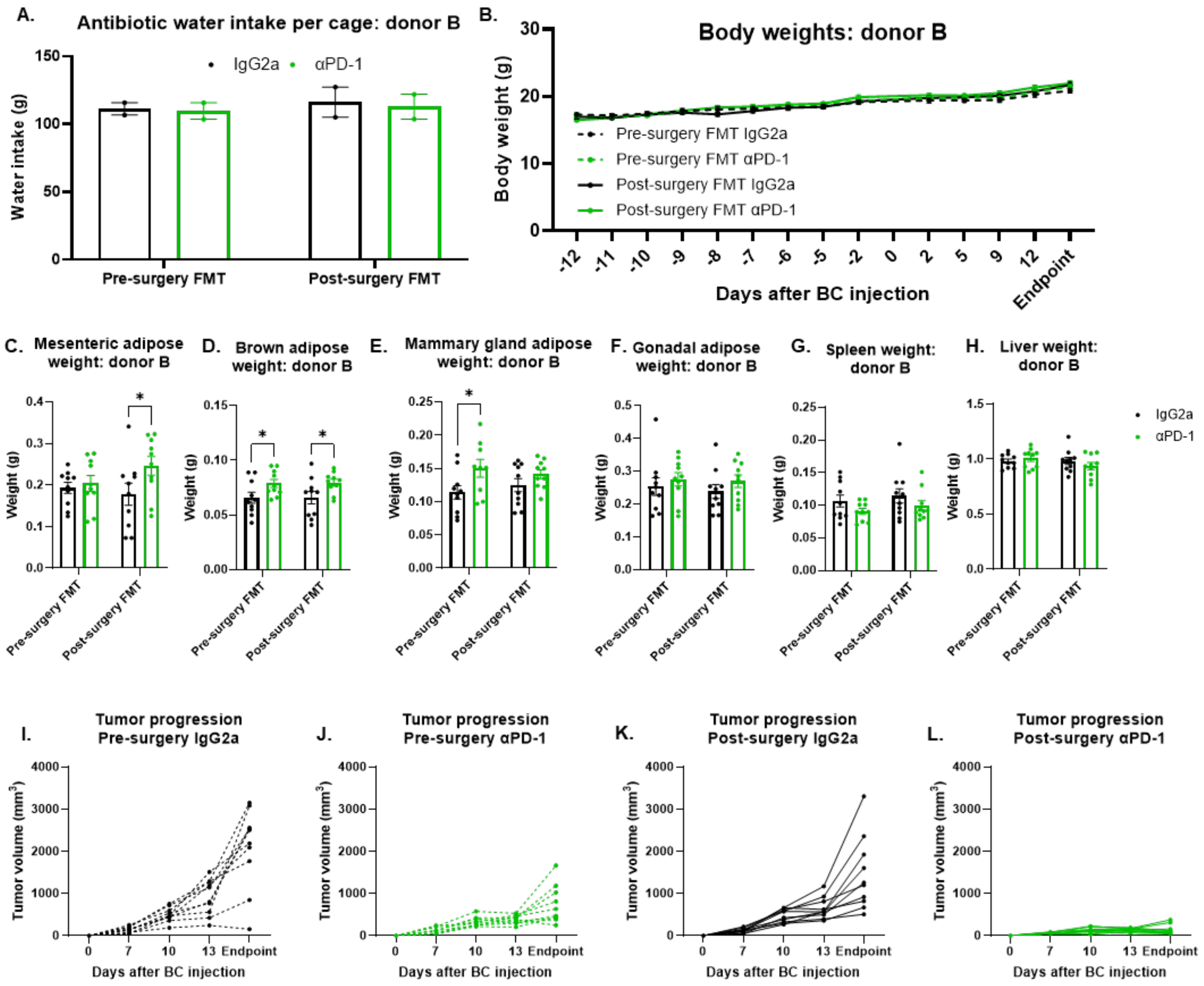

Supplemental Figure 5. Microbes from Donor B pre- and post-surgery do not impact body weight of recipients.

(**A**) Antibiotic water intake was measured as mass in grams at the cage level over the course of the entire 8-day antibiotic treatment. All cages received antibiotic cocktail. N = 2 cages per group, with N=5 mice per cage. (**B**) Body weights were measured over the duration of the study. Significance was determined by two-way ANOVA mixed effects model with Fisher's LSD test; N = 10 per group. **C-F**) Adipose tissue weights were measured as mass in grams for mesenteric (**C**), brown (**D**), mammary (**E**), and gonadal adipose (**F**) depots. Data are presented as mean ± SEM with comparisons determined by two-way ANOVA. N = 10 per group. (**G-H**) Organ weights were measured as mass in grams for spleen (**G**) and liver (**H**). Data are presented as mean ± SEM with comparisons determined by two-way ANOVA. N = 10 per group. (**I-L**) Tumor progression in individual mice in each treatment group from Figure 2F, including pre-surgery FMT IgG2a (**I**), pre-surgery FMT αPD-1 (**J**), post-surgery FMT IgG2a (**K**), and post-surgery FMT αPD-1 (**L**); N = 10 per group. *p<0.05.

Supplemental Figure 6

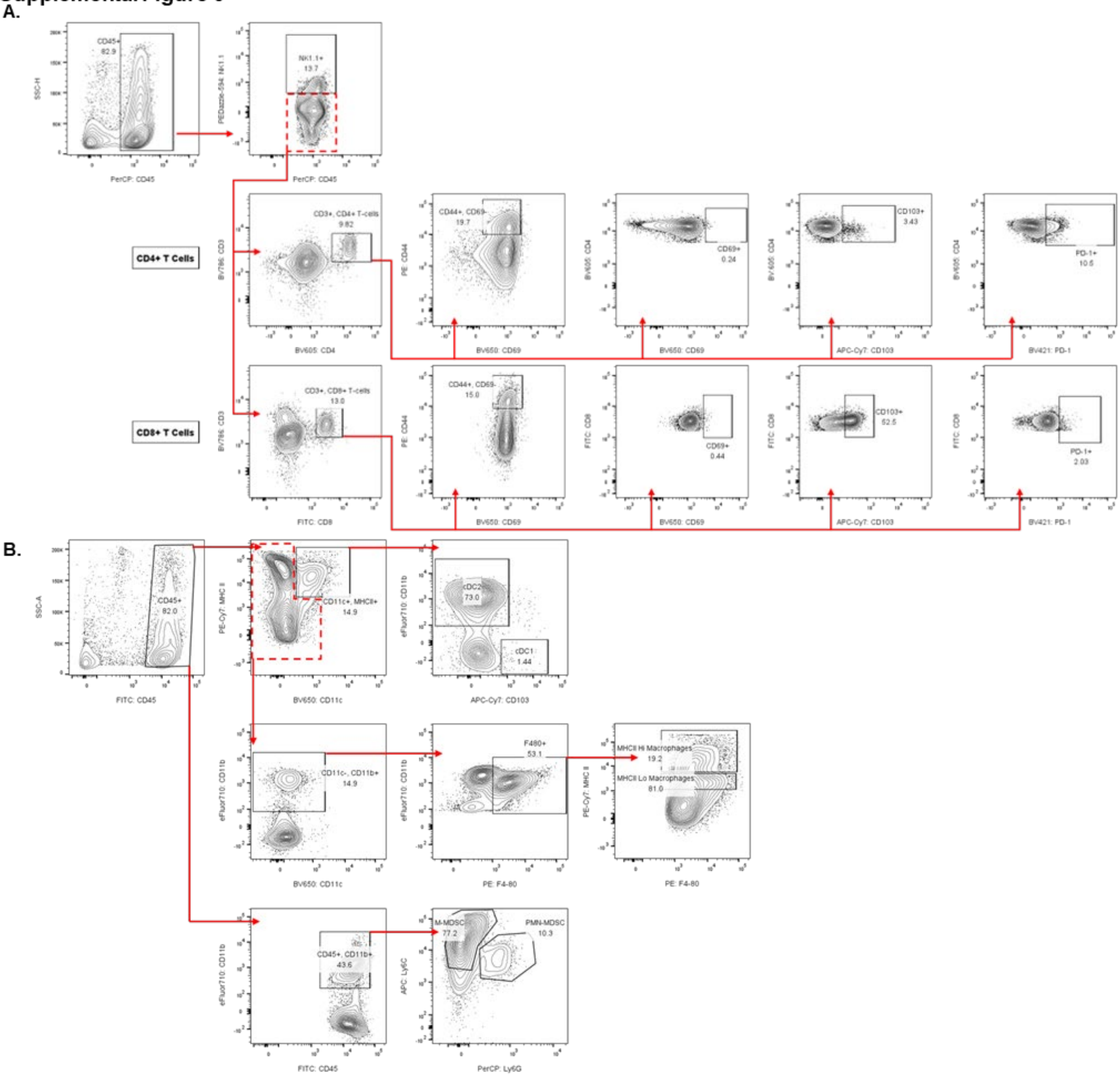

Supplemental Figure 6. Tumor Gating Schemes.

(**A**) Flow cytometric gating scheme for T cell panel used on tumor tissues collected from mouse-to-mouse FMT study described in Figure 1A. (**B**) Flow cytometric gating scheme for myeloid panel used on tumor tissues collected from mouse-to-mouse FMT study described in Figure 1A.

Supplemental Figure 7

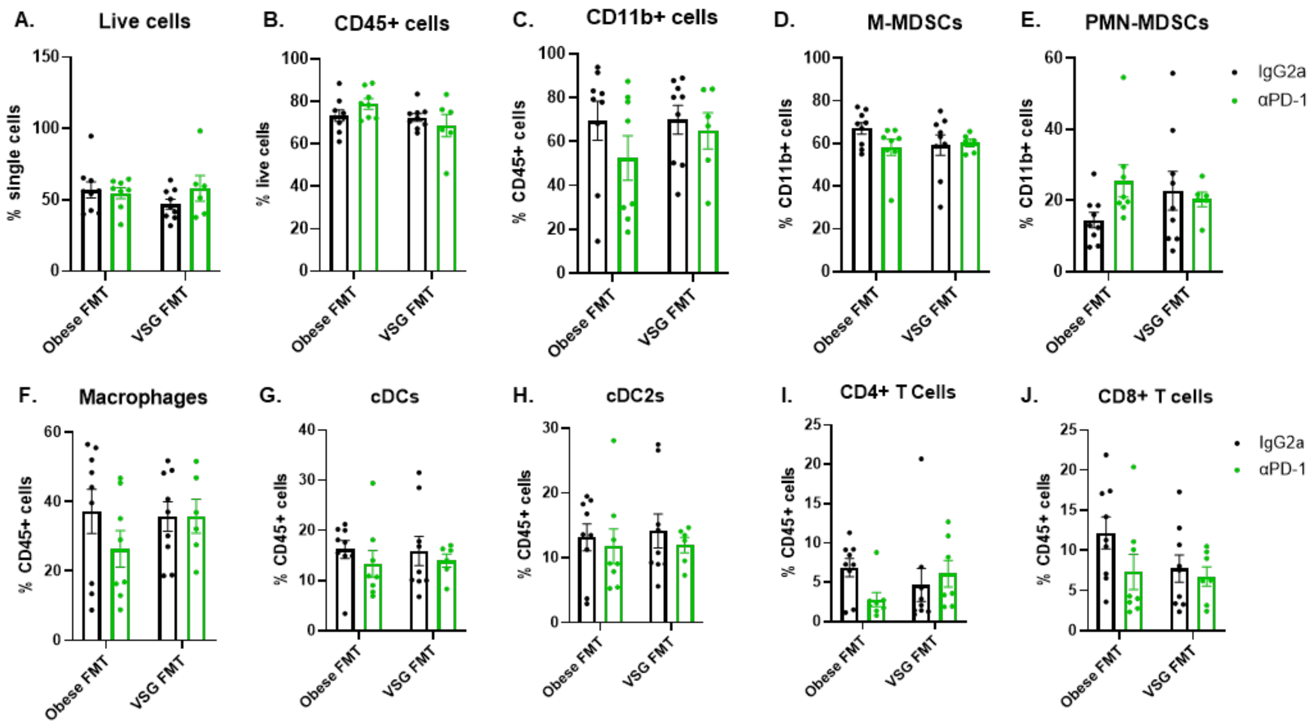

Supplemental Figure 7. Microbes from VSG donors did not demonstrate significant impacts on the tumor immune content.

Flow cytometry analysis of immune cell populations in the tumor immune microenvironment (TIME) is shown as frequency of live cells out of single cells (**A**), frequency of CD45+ cells out of live cells (**B**), frequency of CD11b+ cells out of CD45+ cells (**C**), frequency of monocytic-myeloid derived suppressor cells (**D**, M-MDSCs, CD11b+, Ly6C/Ly6G-), polymorphonuclear-MDSCs (**E**, PMN-MDSCs, CD11b+, Ly6C low/Ly6G+), and macrophages (**F**, CD11b+, Ly6C/Ly6G-, F480+) out of CD11b+ cells, frequency of conventional dendritic cells (**G**, cDCs, CD11c/MHC II+), cDC2s (**H**, CD11c/MHC II+, CD11b high/CD103 low), CD4+ T cells (**I**, CD3+, CD4+) and CD8+ T cells (**J**, CD3+, CD8+) out of CD45+ cells. Data are presented as mean ± SEM with two-way ANOVA analyses; N = 7-8 per group.

Supplemental Figure 8

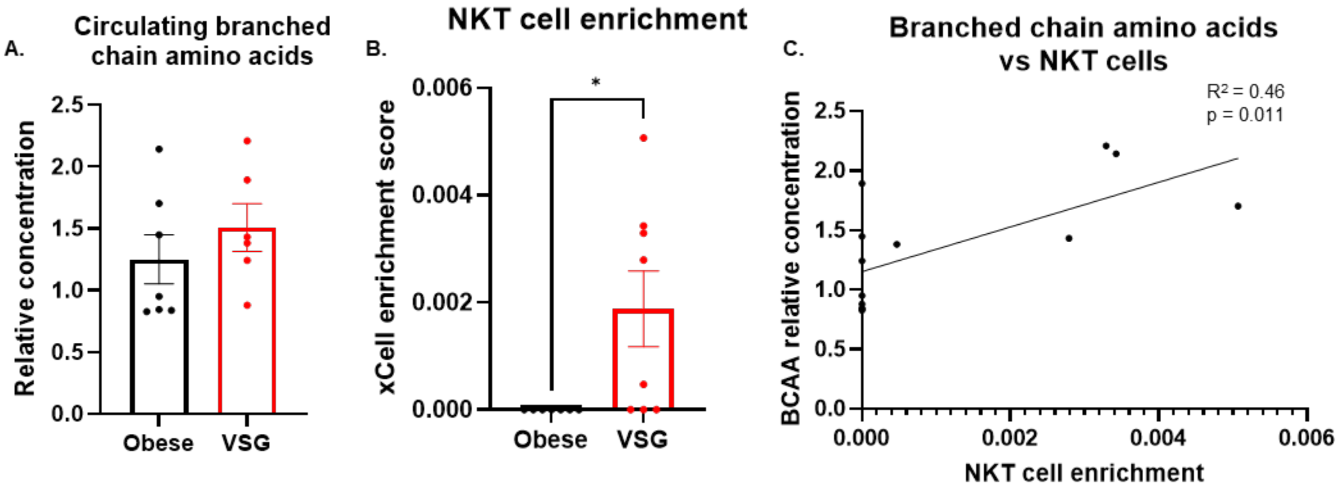

Supplemental Figure 8. In donor cecal content, vertical sleeve gastrectomy modulated the microbially derived metabolite profile and elevated intra-tumoral NKT cells.

(**A**) Relative concentration of circulating branched chain amino acids (BCAA, sum of valine, leucine, and isoleucine) in FMT donors quantified by GC-MS analysis of plasma samples is presented as mean ± SEM with Student's *t*-test analysis. N = 6-7 per group. (**B**) NKT cell enrichment within the TIME of FMT donors calculated using xCell algorithm in Timer2.0 in bulk RNAseq data. Significant comparisons determined by Student's *t*-test; N = 7 – 8. *p<0.05. (**C**) Circulating BCAA concentrations from FMT donors was correlated with NK T cell enrichment scores within the TIME via linear regression analysis. R^2 = 0.46, p = 0.011, N = 11.

Supplemental Figure 9

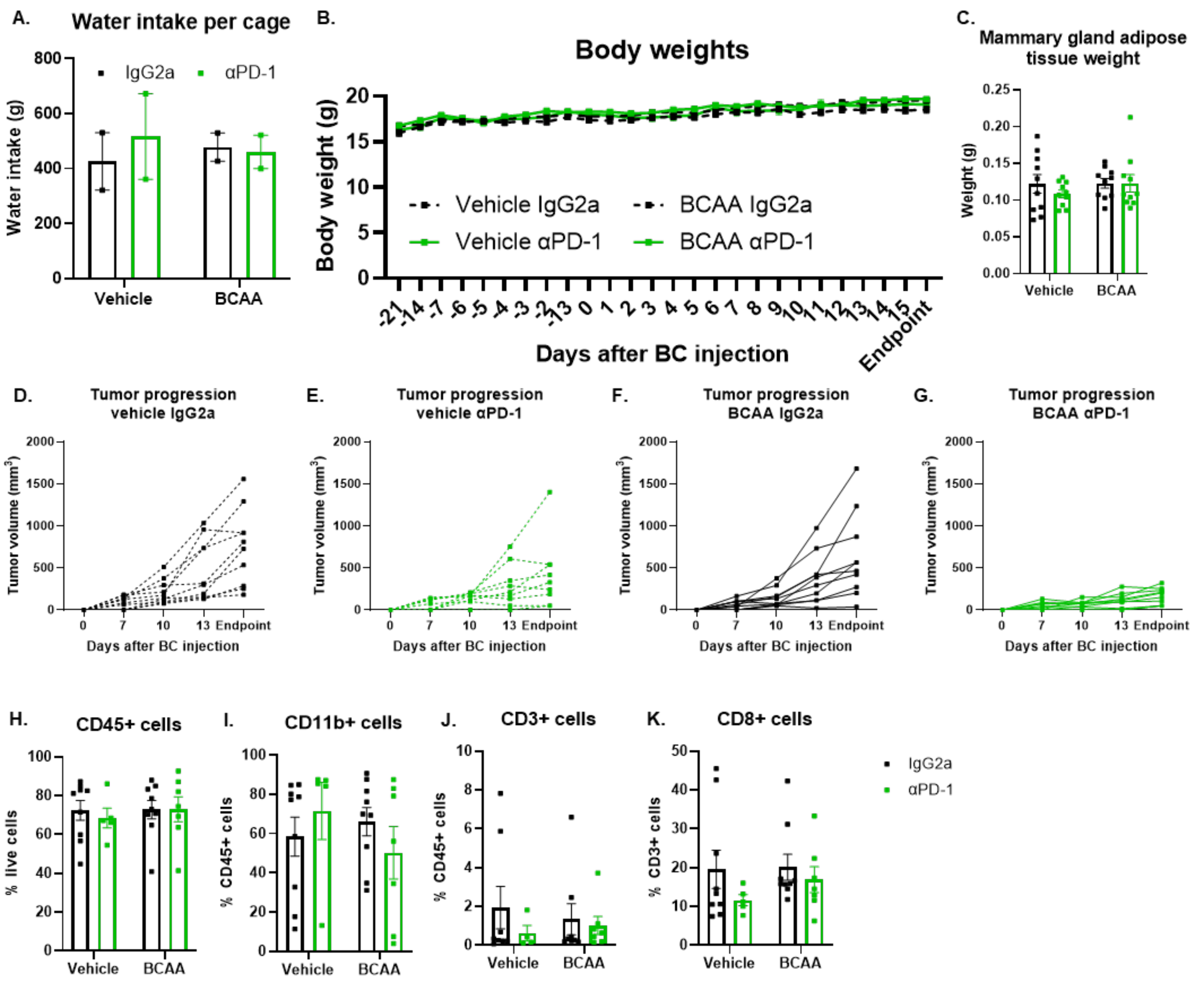

Supplemental Figure 9. Branched chain amino acid supplementation did not impact the body weight of recipients in E0771 model.

(**A**) Branched chain amino acid (BCAA) water intake was measured as mass in grams at the cage level over the course of the entire 22-day supplementation. Vehicle cages received regular autoclaved drinking water. N = 2 cages per group, with N=5 mice per cage. (**B**) Body weights were measured over the duration of the study. Significance was determined by two-way ANOVA mixed effects model with Fisher's LSD test; N = 10 per group. (**C**) Mammary gland adipose tissue depot weight was measured as mass in grams. Data presented as mean ± SEM with comparisons determined by two-way ANOVA. N = 10 per group. (**D-G**) Tumor progression in individual mice in each treatment group from Figure 5B, including vehicle IgG2a (**D**), vehicle αPD-1 (**E**), BCAA IgG2a (**F**), and BCAA αPD-1 (**G**); N = 10 per group. (**H-K**) Flow cytometry analysis of immune cell populations in tumor immune microenvironment (TIME) is shown as frequency of CD45+ cells our of live cells (**H**), frequency of CD11b+ cells out of CD45+ cells (**I**), frequency of CD3+ T cells out of CD45+ cells (**J**), and

frequency of CD8+ T cells out of CD3+ T cells (**K**). Data are presented as mean ± SEM with two-way ANOVA analyses; N = 5-9 per group.

Supplemental Figure 10

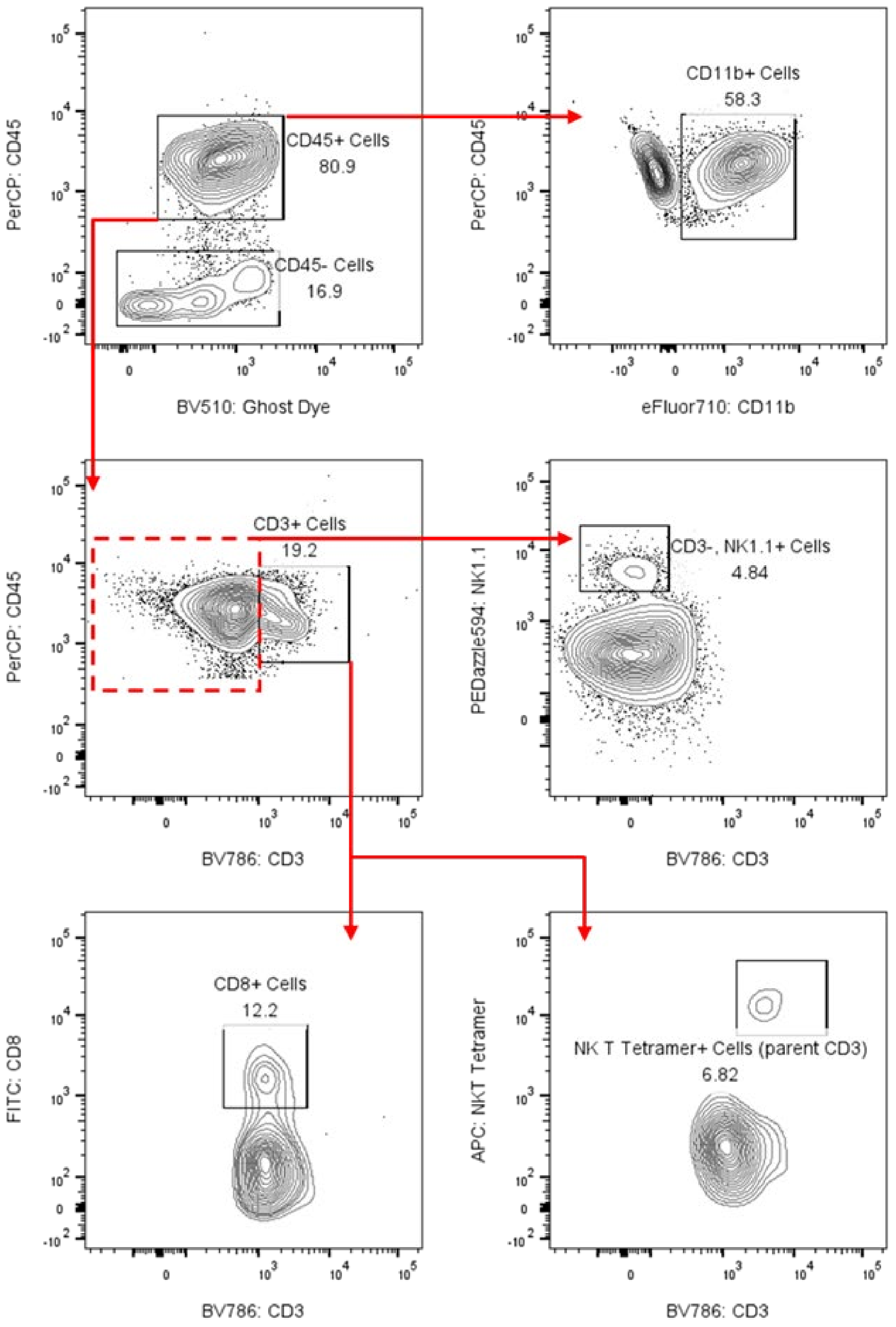

Supplemental Figure 10. Tumor Gating Scheme.

Flow cytometric gating scheme for general panel used on tumor tissues collected from BCAA supplementation study in E0771 model described in Figure 5A.

Supplemental Figure 11

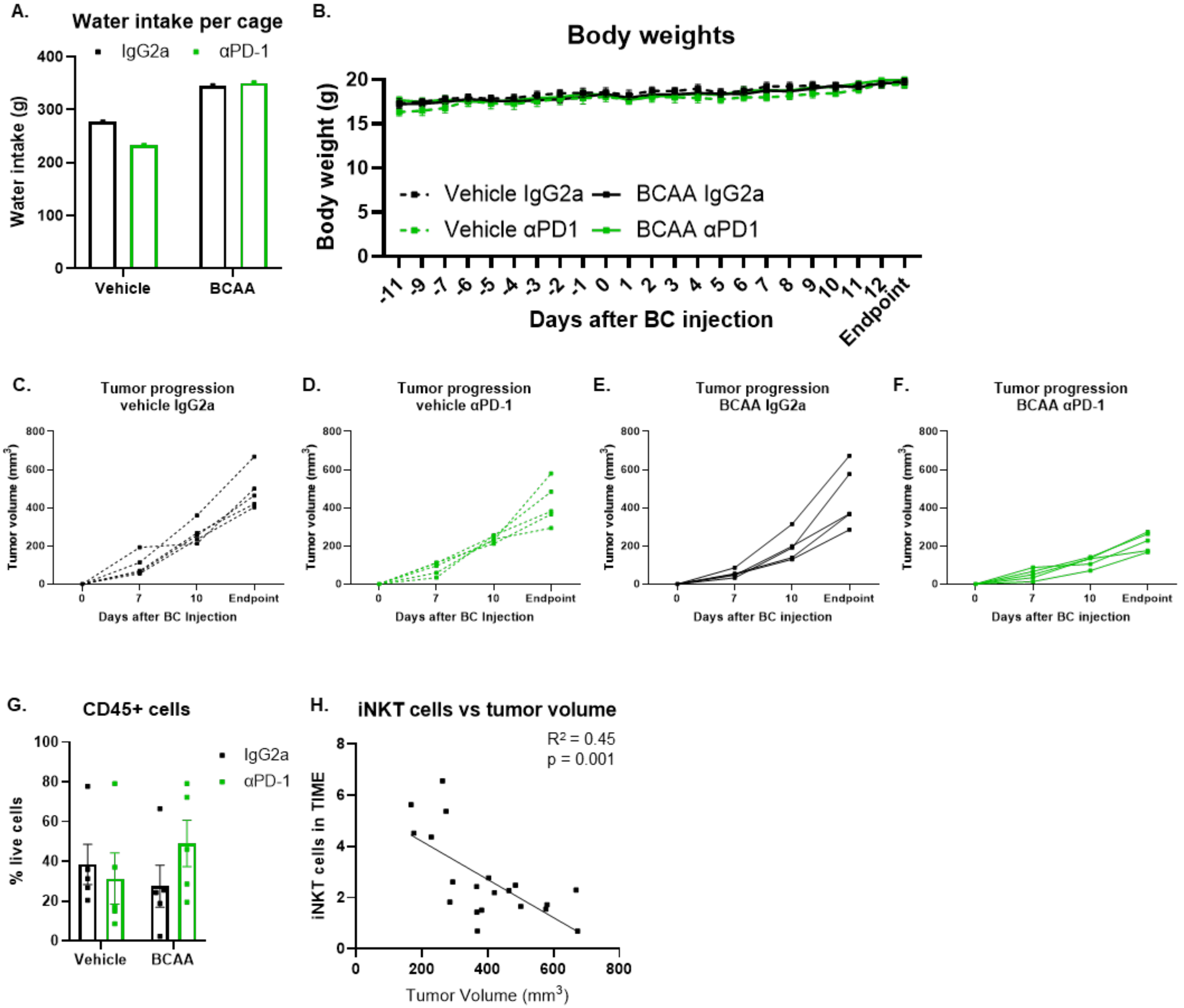

Supplemental Figure 11. Branched chain amino acid supplementation did not impact the body weight of recipients in 4T1 model.

(**A**) Branched chain amino acid (BCAA) water intake was measured as mass in grams at the cage level over the course of the entire 20-day supplementation. Vehicle cages received regular autoclaved drinking water. N = 1 cage per group, with N=5 mice per cage. (**B**) Body weights were measured over the duration of the study. Significance was determined by two-way ANOVA mixed effects model with Fisher's LSD test; N = 5 per group. (**C-F**) Tumor progression in individual mice in each treatment group from Figure 5G, including vehicle IgG2a (**C**), vehicle αPD-1 (**D**), BCAA IgG2a (**E**), and BCAA αPD-1 (**F**); N = 5 per group. (**G**) Flow cytometry analysis of immune cell populations in tumor immune microenvironment (TIME) is shown as frequency of CD45+ cells out of live cells. Data are presented as mean ± SEM with two-way ANOVA analyses; N = 5 per group. (**H**)

Percentage of iNKT cells out of total CD45+ cells within 4T1 TIME were correlated with tumor volume at endpoint via linear regression analysis. R^2 = 0.45, p = 0.001, N = 20.

Supplemental Figure 12

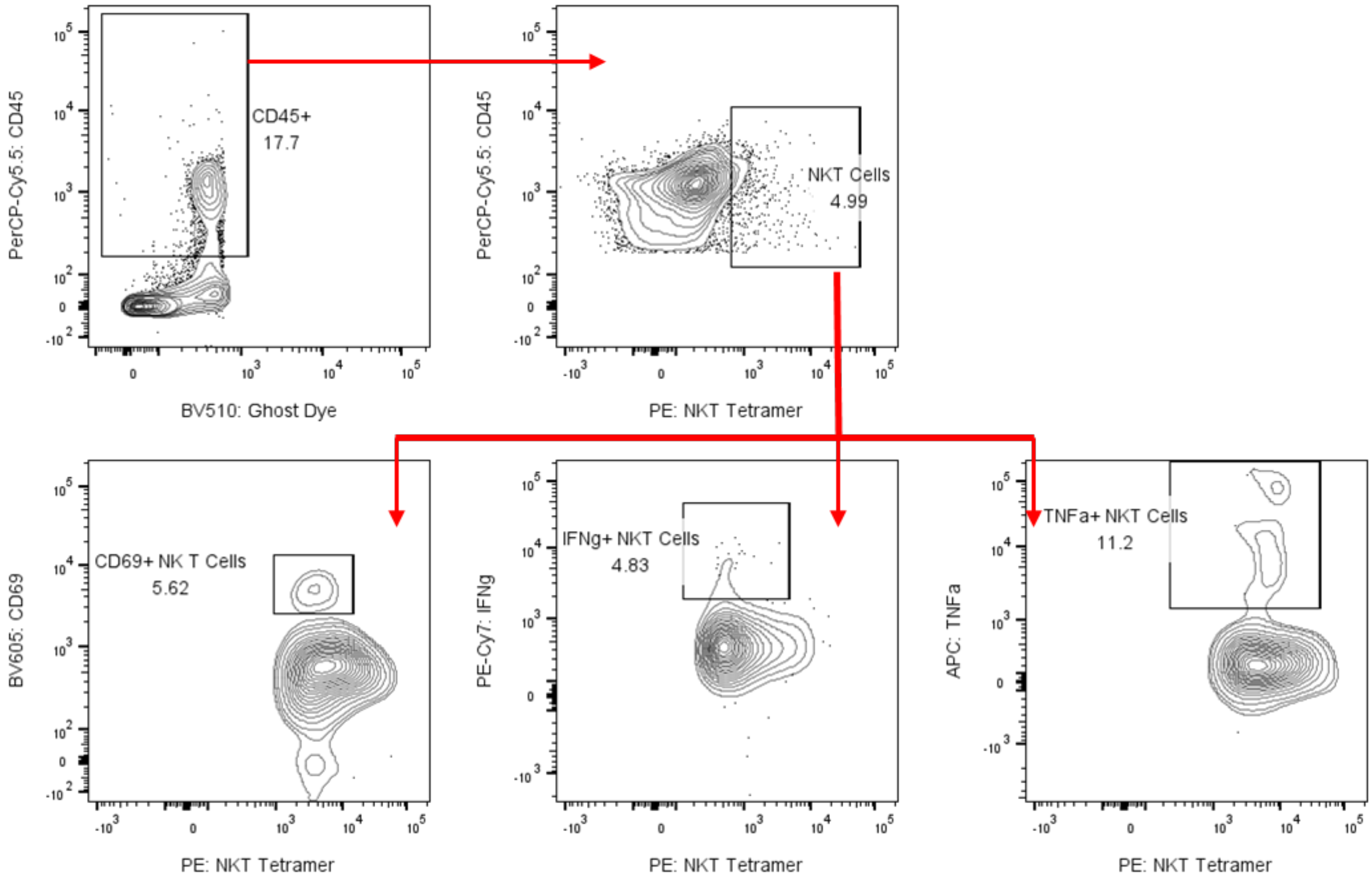

Supplemental Figure 12. Tumor Gating Scheme.

Flow cytometric gating scheme for NKT cell panel used on tumor tissues collected from BCAA supplementation study in 4T1 model described in Figure 5A.

Supplemental Figure 13

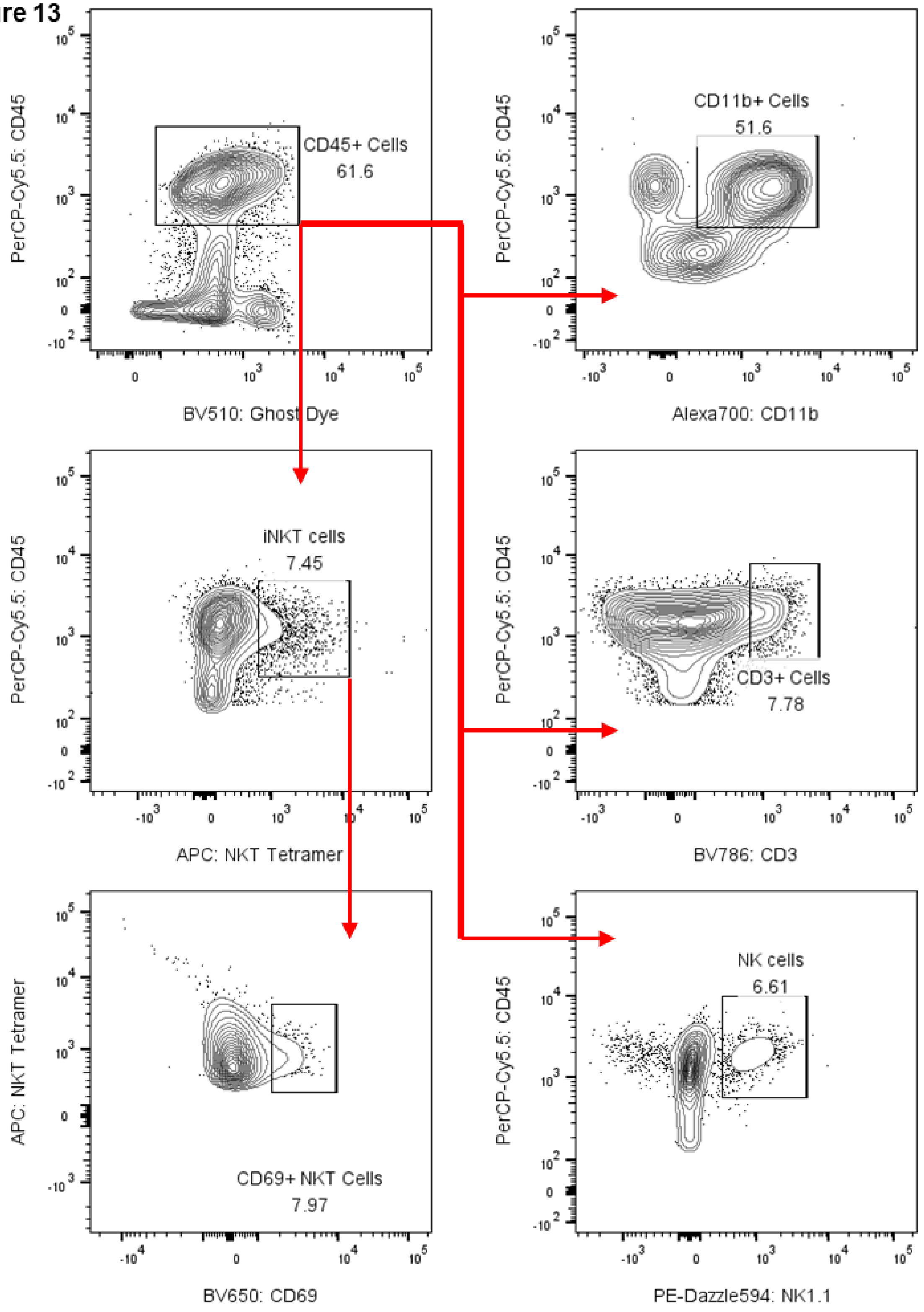

Supplemental Figure 13. Tumor Gating Scheme.

Flow cytometric gating scheme for NKT cell panel used on tumor tissues collected from Donor B human-to-mouse FMT study described in Figure 2A.

Supplemental Figure 14

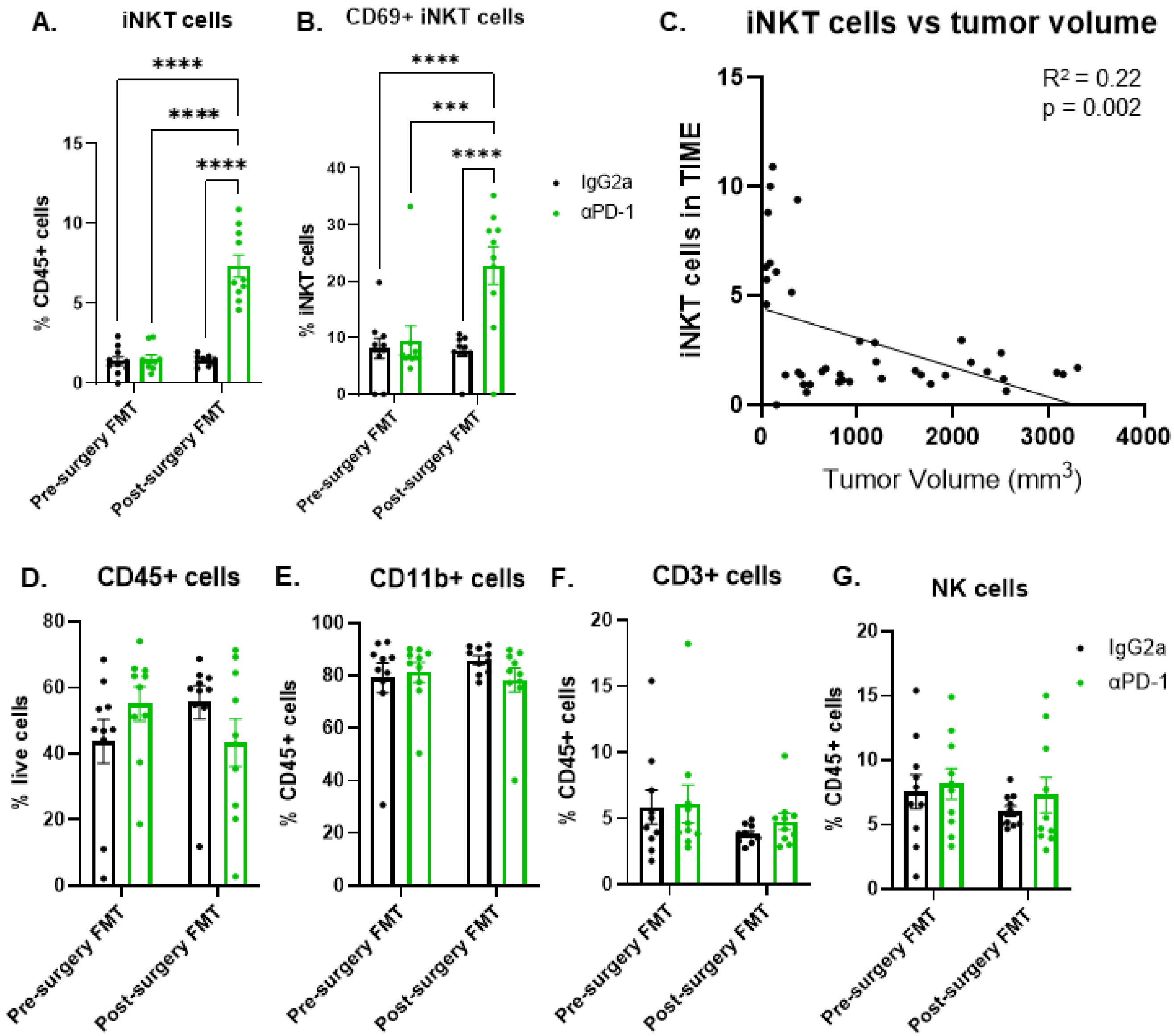

Supplemental Figure 14. Post-surgery FMT from Donor B increases iNKT cells in the TIME.

(**A-B**) Flow cytometry analysis of immune cell populations in tumor immune microenvironment (TIME) from human-to-mouse FMT Donor B in Figure 2 is shown as frequency of iNKT cells out of total CD45+ cells (**A**) and frequency of CD69+ iNKT cells out of total iNKT cells (**B**). Data are presented as mean ± SEM with two-way ANOVA analyses; N = 10 per group. (**C**) Percentage of iNKT cells out of total CD45+ cells within TIME were correlated with tumor volume at endpoint via linear regression analysis. $R^2 = 0.22$, p = 0.002, N = 40. (**D-G**) Flow cytometry analysis of immune cell populations in TIME is shown as frequency of CD45+ cells our of live cells (**D**), frequency of CD11b+ cells out of CD45+ cells (**E**), frequency of CD3+ T cells out of CD45+ cells (**F**), and frequency of NK cells out of CD45+ cells (**G**). Data are presented as mean ± SEM with two-way ANOVA analyses; N = 10 per group. ***p<0.001, ****p<0.0001.

Supplemental Figure 15

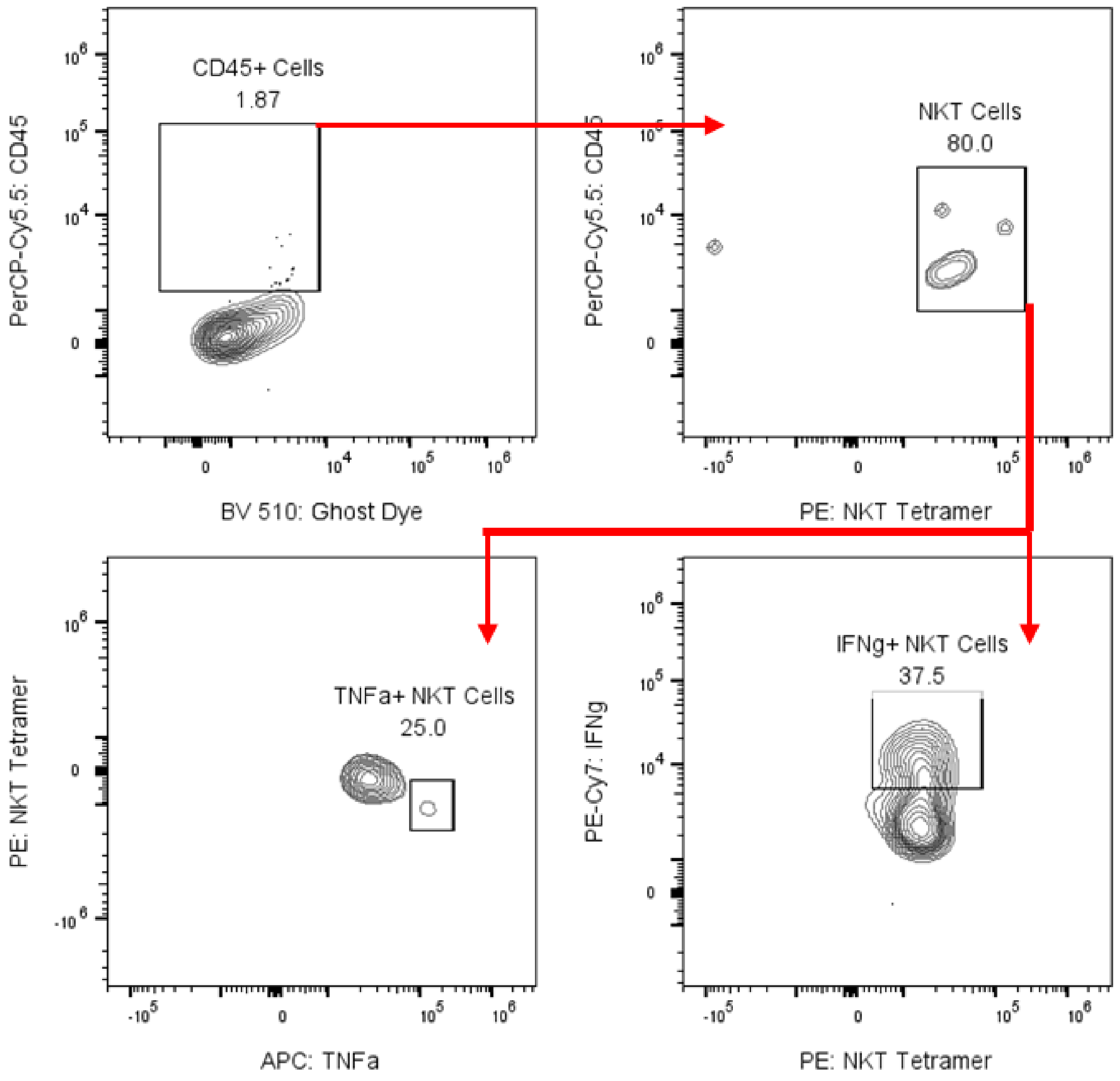

Supplemental Figure 15. Tumor Gating Scheme.

Flow cytometric gating scheme for NKT cell panel used on primary iNKT cells described in Figure 6A.

**Supplementary Table 3.** Antibodies for flow cytometric analysis of tumor immune microenvironment.			
Antibody	Source	Identifiers	Dilutions
Anti-Human/Mouse CD11b redFluor 710	Tonbo	80-0112-U025	1:20
APC anti-mouse Ly-6C	BioLegend	128015	1:40
Anti-Mouse F4/80 Antigen PE	Tonbo	50-4801-U025	1:40
Anti-Mouse Ly-6G PerCP-Cyanine5.5	Tonbo	65-1276-U025	1:40
Brilliant Violet 650 anti-mouse CD11c	BioLegend	117339	1:10
PE/Cyanine7 anti-mouse I-A/I-E (MHC class II)	BioLegend	107630	1:40
FITC anti-mouse CD45	BioLegend	103108	1:40
Brilliant Violet 711 anti-mouse CD274 (B7-H1,PD-L1)	BioLegend	124319	1:10
APC/Cyanine7 anti-mouse CD103	BioLegend	121432	1:5
Anti-Mouse CD8a FITC	Tonbo	35-0081-U025	1:10
Brilliant Violet 605 anti-mouse CD4	BioLegend	100548	1:10
Brilliant Violet 785 anti-mouse CD3ε	BioLegend	100355	1:4
PE/Dazzle 594 anti-mouse NK-1.1	BioLegend	108747	1:10
Brilliant Violet 421 anti-mouse CD279 (PD-1)	BioLegend	109121	1:10
PerCP/Cyanine5.5 anti-mouse CD45	BioLegend	103132	1:20
PE anti-mouse/human CD44	BioLegend	103008	1:20
Brilliant Violet 650 anti-mouse CD69	BioLegend	104541	1:05
Alexa Fluor 700 anti-mouse CD62L	BioLegend	104426	1:40
APC anti-mouse iNKT Tetramer	NIH Tetramer Core	PBS57-CD1d	1:5
PE anti-mouse iNKT Tetramer	NIH Tetramer Core	PBS57-CD1d	1:5
PE/Cyanine 7 anti-mouse IFN-γ	BioLegend	505825	1:5
APC anti-mouse TNF-α	BioLegend	506308	1:5

Supplemental Methods for Bohm et al. The gut microbiome enhances breast cancer immunotherapy following bariatric surgery

Reagents

All reagents were obtained from Sigma-Aldrich unless otherwise noted. Fetal bovine serum (FBS, Gibco), 100x GlutaMAX (Gibco), and 100x Penicillin/Streptomycin (HyClone) were purchased from ThermoFisher. RPMI 1640 and Matrigel are from Corning. Rodent diets were obtained from Research Diets Inc. With the exception of vancomycin (V2002 Sigma-Aldrich), antibiotics were purchased from Research Products International including neomycin (N20040), ampicillin (A40040), and cefoperazone (C57600). In vivo anti-mouse PD-1 monoclonal antibody (clone RMP1-14, BE0146) and control immunoglobulin (rat IgG2a, clone 2A3, BE0089) were obtained from BioxCell. Flow cytometry antibodies (Supplemental Table 3), compensation beads, and reagents are from Tonbo Biosciences, Inc. and Biolegend. BCAAs were purchased from Sigma-Aldrich, including leucine (L8000), isoleucine (I2752), and valine (V0513).

Animals, Diet, and Antibiotic Exposure

Mice were randomized into 5 mice per cage. All animals were housed in a temperature-controlled facility with a 12-hour light/dark cycle and *ad libitum* access to purified defined diets as follows. Low fat diet (LFD) with 10% kcal from fat (D12450Ji Research Diets Inc.) was introduced 24 hours after arrival at our facility and mice remained on this diet for the duration of the study. LFD was chosen because donors were fed high fat diet (HFD) with 60% kcal from fat (D12492i Research Diets Inc.), and LFD is matched in micronutrients to the HFD (1, 2). Mice were housed for 2 weeks prior to intervention and bedding was mixed between cages weekly to normalize baseline microbiomes across cages to reduce cage effects.

Fecal Microbial Transplant Donors and Sample Preparation

Sipe et *al*. used specific pathogen free C57BL/6J females that were group housed and were fed either LFD to remain lean or made obese by HFD exposure for 16 weeks. In that study, all mice received a sham surgery except one obese group that received VSG to induce weight loss with surgeries conducted as previously published (1) and all mice remained on HFD. In brief, surgeries were performed under isoflurane

anesthesia. In VSG group, the stomach was clamped and 80% of the stomach was removed. The remaining stomach was sutured to create a tubular gastric sleeve. Obese control group had a sham surgery wherein the abdominal laparotomy was performed, and light pressure was applied to the stomach with forceps. Mice were closed with sutures and received carprofen as an analgesic immediately prior to and for 3 days following surgery. Upon stable weight loss after VSG or maintenance of obesity in sham controls, mice were injected orthotopically with E0771 BC cells to examine BC progression, which demonstrated that VSG led to reduced tumor progression compared to obese sham controls (1). At endpoint, cecal contents were collected for use in this study presented herein.

Fecal slurry was prepared from cecal contents from Sipe et *al*. mice (N = 5-17 mice pooled per group) (1). Cecal contents were resuspended at 20-30 mg/mL in glycerol/PBS with reduced oxygen under anaerobic conditions. The slurry was aliquoted using a cut 200 μL tip and stored at -80°C. An aliquot from each group was used for quality control via 16S sequencing at University of Wisconsin Biotechnology Center DNA Sequencing Facility. Aliquots were thawed 1 hour prior to gavage on wet ice for FMTs.

Fecal Microbial Transplant

There were two control groups: control (no antibiotics or gavage) and antibiotics only (no gavage). Three experimental groups included FMT gavage from the following donors: lean + sham (lean FMT), obese + sham (obese FMT), and VSG bariatric surgery (VSG FMT). To ensure engraftment of microbiota from lean or obese donors accustomed to a specific diet (2), for 24 hours prior to and 24 hours following the first FMT, mice receiving microbes from donors that were previously on HFD were switched from LFD to HFD with 60% kcal from fat (D12492i Research Diets, Inc.) including obese FMT and VSG FMT groups, while lean FMT recipients remained on LFD to match the donor diet. After the first FMT, all mice were maintained on LFD to minimize alterations to body weight. After the first two FMT gavages, mice were orthotopically injected with the E0771 BC cell line (1, 3), then another two FMT gavages were subsequently administered (Figure 1A).

Human Fecal Collection and Sample Preparation

The patients lost 24% and 13%, respectively, of starting body weight by the post-surgery collection timepoint (Figure 2A) with pre-surgery BMI of 41.6 and 50.3, respectively, and post-surgery BMI of 31.4 and

44.6, respectively. Researchers were blinded to patient identities. Deidentified samples were resuspended at 25 mg/mL in glycerol/PBS with reduced oxygen under anaerobic conditions. Slurry was aliquoted using a cut 200 µL tip and stored at -80°C. An aliquot from each group was used for quality control via 16S sequencing. Aliquots were thawed 1 hour prior to gavage on wet ice for FMT, as described above.

After 8 days of antibiotic cocktail, mice received pre-surgery FMT gavage (n=20) or post-surgery FMT gavage (n=20). Following two gavages, mice were injected with E0771 cells as above, followed by two additional FMT gavages. ICB or IgG2a isotype control were administered as described below. Upon endpoint, tissues were collected as described below.

Cell Culture and Tumor Implantation

Cells tested negative for *Mycoplasma* (LT07-318 Lonza). Briefly, cells were cultured in RPMI 1640 containing 10% FBS, 2 mM GlutaMAX, 100 UI/mL penicillin, and 100 µg/mL streptomycin in a humidified chamber at 37°C under 5% CO_2. E0771 cells were orthotopically injected in the left fourth mammary fat pad of 8-week-old mice at 250,000 cells in 100 µL of 25% Matrigel Matrix High Concentration (354248 Corning), 75% RPMI with a final Matrigel concentration of 5 mg/mL. 4T1 cells were also orthotopically injected in the left fourth mammary fat pad of 8-week-old mice at 500,000 cells in 100 µL of 25% Matrigel Matrix High Concentration (354248 Corning), 75% RPMI with a final Matrigel concentration of 5 mg/mL. Starting seven days after implantation, tumor growth was monitored every third day by measuring the length and width of the tumor using digital calipers. Tumor volume was calculated using the following formula: $\text{Volume} = (\text{width})^2 \times (\text{length})/2$ (1). No tumors failed to take in any of the studies. At experimental endpoint (days 13-20 after tumor cell injection), excised tumor mass was determined.

Immune Checkpoint Blockade

Following BC cell injection, immune checkpoint blockade αPD-1 antibody (clone RMP1-14, BE0146 BioxCell) or rat IgG2a isotype control (clone 2A3, BE0089 BioxCell) were administered at 200 µg per mouse in 50 µL PBS by intraperitoneal injection. Antibody administration began 3 days after BC cell implantation. ICB or control were administered every 3 days until endpoint (3).

Tissue and Blood Collection

At experimental endpoint, mice were fasted 4 hours prior to isoflurane anesthesia. Blood was collected via cardiac puncture into EDTA-coated syringes. Plasma was separated from other blood components by centrifugation at 1200 x g for 45 min at 10°C. Mammary tumors, unaffected mammary fat pads from the contralateral side, gonadal adipose, mesenteric adipose (surrounding intestine), intestinal microbiota contents, spleen, liver, and brown adipose were weighed and either snap frozen in liquid nitrogen or digested into a single cell suspension for flow cytometry. All frozen samples were stored at -80°C until analyzed.

RNA Isolation and Sequencing

Fastq files obtained from Illumina HiSeq underwent quality control assessment via FastQC (version 0.1.3 (4)). Files were first aligned to the mouse transcriptome (mm10/GRCm38.p4 genome build with Ensembl v86 gene annotation) using STAR (version 0.6-5 (5)). Aligned reads were sorted by SAMtools (version 1.6.4 (6)) and sorted reads were subjected to the quantification via Salmon (7). ENSEMBL gene level counts were used for data analysis in R version 4.3.1. Read counts loaded from Salmon quant files via tximport (8) were subjected to differential gene expression (DEG) analysis between two groups in four comparisons via DESeq2 (9). Pseudogenes and RIKEN genes identified were removed. An adjusted p-value < 0.1 was used to determine DEGs from each sample group. Read counts were normalized for downstream analyses and visualization using the regularized log normalization (rlog) from DESeq2.

Gene set enrichment analysis (GSEA)

For identification of enriched gene signatures, we used GSEA software (10). GSEA analysis was performed by using rlog-normalized gene expression data obtained from treatment groups. A 1,000 gene set permutation parameter was used to test for significance at a false discovery rate (FDR) threshold of 0.25. The mouse MSigDB hallmark gene sets (MH collection (11)) were used to determine enriched pathways in αPD-1 and IgG groups as well as VSG FMT and obese FMT groups. Normalized enrichment scores (NES) are reported. For GSEA hallmark gene sets, nominal p-value was less than 0.05 were shown. GSEA plots were generated by ggplot2 R package (version 3.5.1 (12)).

Microbiota Analysis

Purified DNA samples underwent 16S library preparation and NextGen Illumina MiSeq sequencing at University of Wisconsin – Madison Bioinformatics Resources Core. Controls included empty tubes passed through an identical collection, extraction, and amplification process but remained free of DNA amplification (data not shown).

Flow Cytometric Analysis of Tumors

Tumor pieces were digested as per mouse tumor dissociation kit instructions and digested tissue was filtered through 70 μm strainer to obtain a single cell suspension. Antibodies used (Supplemental Table 3) were titrated and the separation index was calculated using FlowJo version 10 software for every study. For Figures 5, 6, and Supplemental Figure 14 specifically, NKT cells were labeled with soluble APC-tagged or PE-tagged PBS57-CD1d tetramers provided by the NIH Tetramer Core Facility to specifically identify iNKT subsets. A minimum number of 1000 events were considered for analysis. Fluorescence minus one (FMO) stained cells and single color Ultracomp Beads (Invitrogen) were used as negative and positive controls, respectively.

Ex Vivo Branched Chain Amino Acid Assays

In brief, lymphocytes were isolated from female C57BL/6J mouse livers via Percoll gradient centrifugation (13) and FACS-sorted using soluble APC-tagged PBS57-CD1d tetramers provided by the NIH Tetramer Core Facility. Sorting yielded populations that were more than 95% PBS57-CD1d Tetramer+.

References

1. Sipe LM, Chaib M, Korba EB, Jo H, Lovely MC, Counts BR, et al. Response to immune checkpoint blockade improved in pre-clinical model of breast cancer after bariatric surgery. *Elife.* 2022;11.
2. Bohm MS, Ramesh AV, Pierre JF, Cook KL, Murphy EA, and Makowski L. Fecal microbiome transfer an investigative tool and treatment strategy in cancer. *Am J Physiol Gastrointest Liver Physiol.* 2024.
3. Pingili AK, Chaib M, Sipe LM, Miller EJ, Teng B, Sharma R, et al. Immune checkpoint blockade reprograms systemic immune landscape and tumor microenvironment in obesity-associated breast cancer. *Cell Rep.* 2021;35(12):109285.
4. Andrews S. FastQC: A Quality Control Tool for High Throughput Sequence Data. http://www.bioinformatics.babraham.ac.uk/projects/fastqc/.
5. Dobin A, Davis CA, Schlesinger F, Drenkow J, Zaleski C, Jha S, et al. STAR: ultrafast universal RNA-seq aligner. *Bioinformatics.* 2013;29(1):15-21.
6. Danecek P, Bonfield JK, Liddle J, Marshall J, Ohan V, Pollard MO, et al. Twelve years of SAMtools and BCFtools. *Gigascience.* 2021;10(2).

7. Patro R, Duggal G, Love MI, Irizarry RA, and Kingsford C. Salmon provides fast and bias-aware quantification of transcript expression. *Nat Methods.* 2017;14(4):417-9.
8. Soneson C, Love MI, and Robinson MD. Differential analyses for RNA-seq: transcript-level estimates improve gene-level inferences. *F1000Res.* 2015;4:1521.
9. Love MI, Huber W, and Anders S. Moderated estimation of fold change and dispersion for RNA-seq data with DESeq2. *Genome Biol.* 2014;15(12):550.
10. Subramanian A, Tamayo P, Mootha VK, Mukherjee S, Ebert BL, Gillette MA, et al. Gene set enrichment analysis: a knowledge-based approach for interpreting genome-wide expression profiles. *Proc Natl Acad Sci U S A.* 2005;102(43):15545-50.
11. Liberzon A, Birger C, Thorvaldsdottir H, Ghandi M, Mesirov JP, and Tamayo P. The Molecular Signatures Database (MSigDB) hallmark gene set collection. *Cell Syst.* 2015;1(6):417-25.
12. Wickham H. *ggplot2: Elegant Graphics for Data Analysis*. Cham: Springer International Publishing; 2016:189-201.
13. Bassiri H, Das R, Guan P, Barrett DM, Brennan PJ, Banerjee PP, et al. iNKT cell cytotoxic responses control T-lymphoma growth in vitro and in vivo. *Cancer Immunol Res.* 2014;2(1):59-69.

Analytical Code for Bohm et al. The gut microbiome enhances breast cancer immunotherapy following bariatric surgery

RNAseq Analysis

```
# useful function for DESeq2 RNAseq analysis
# Jeremiah Holt


library(tidyverse)
library(ggplot2)
library(ggrepel)
library(EnhancedVolcano)
library(RColorBrewer)
library(gridExtra)
library(org.Mm.eg.db)

############################
##### plotDistribution #####
############################
# function that gathers count matrix (rows = genes, cols = samples)
# and attaches colData for box, density, or violin plotting in ggplot2.

plotDistribution = function(dds, plot_type="boxplot", norm=FALSE) {
  if(norm == TRUE){
    long.df = as.data.frame(log2(counts(dds, normalized=T)+1)) %>% # only normalized for library size
      mutate(gene=rownames(.), .before=1) %>%
      tidyr::pivot_longer(!gene, names_to="sample", values_to="log2Expression") %>%
      inner_join(as.data.frame(colData(dds)), by="sample") %>%
      dplyr::select(!file) %>%
      relocate(c(treatment, group), .after=sample)

  }else{
    long.df = as.data.frame(log2(counts(dds)+1)) %>%
```

```
    mutate(gene=rownames(.), .before=1) %>%
    tidyr::pivot_longer(!gene, names_to="sample", values_to="log2Expression") %>%
    inner_join(as.data.frame(colData(dds)), by="sample") %>%
    dplyr::select(!file) %>%
    relocate(c(treatment, group), .after=sample)
}

# change sample order for factors on x axis
order = long.df %>%
  arrange(treatment, group) %>%
  distinct(sample) %>%
  pull(sample)



if (plot_type == "boxplot") {
  p = long.df %>%
    mutate(sample=factor(sample, levels=order)) %>% # reorder factor levels
    ggplot(aes_string(x = "sample", y = "log2Expression")) +
    geom_boxplot(aes_string(col = "treatment", fill = "group"), alpha = 0.5) +
    theme_bw()
}

if (plot_type == "violin") {
  p = long.df %>%
    mutate(sample=factor(sample, levels=order)) %>% # reorder factor levels
    ggplot(aes_string(x = "sample", y = "log2Expression")) +
    geom_violin(aes_string(col = "treatment", fill = "group"), alpha = 0.5) +
    theme_bw()
}

if (plot_type == "density") {
  p = long.df %>%
    mutate(sample=factor(sample, levels=order)) %>% # reorder factor levels
```

```
    ggplot(aes_string(x = "log2Expression")) +
    geom_density(aes_string(col = "treatment", linetype = "group"), alpha = 0.1) +
    theme_bw()
  }
  return(p)
}

# use this for object that has been VST normalized with DESeq2
plotDistributionNorm = function(vsd, plot_type="boxplot"){
  long.df = as.data.frame(assay(vsd)) %>%
   mutate(gene=rownames(.), .before=1) %>%
   tidyr::pivot_longer(!gene, names_to="sample", values_to="vstLog2Expression") %>%
   inner_join(as.data.frame(colData(dds)), by="sample") %>%
   dplyr::select(!file) %>%
   relocate(c(treatment, group), .after=sample)



  # change sample order for factors on x axis
  order = long.df %>%
   arrange(treatment, group) %>%
   distinct(sample) %>%
   pull(sample)



  if (plot_type == "boxplot") {
   p = long.df %>%
    mutate(sample=factor(sample, levels=order)) %>% # reorder factor levels
    ggplot(aes_string(x = "sample", y = "vstLog2Expression")) +
    geom_boxplot(aes_string(col = "treatment", fill = "group"), alpha = 0.5) +
    theme_bw()
  }

  if (plot_type == "violin") {
```

```
    p = long.df %>%
      mutate(sample=factor(sample, levels=order)) %>% # reorder factor levels
      ggplot(aes_string(x = "sample", y = "vstLog2Expression")) +
      geom_violin(aes_string(col = "treatment", fill = "group"), alpha = 0.5) +
      theme_bw()
  }

  if (plot_type == "density") {
    p = long.df %>%
      mutate(sample=factor(sample, levels=order)) %>% # reorder factor levels
      ggplot(aes_string(x = "vstLog2Expression")) +
      geom_density(aes_string(col = "group", linetype = "treatment"), alpha = 0.1) +
      theme_bw()
  }
  return(p)

}



#######################
##### plotVolcano #####
#######################
# function that takes DEG matrix and plots
# log2FC and -logPvalue in ggplot2.

plotVolcano = function(dif, genes, title, FC=0.01, pvalue.adj=0.8, label=T){
  cutoff.FC=FC
  cutoff.pvalue=pvalue.adj

  # axis limits
  xlim = 10
  ylim = 20
```

```
# add a column for FC and pvalue differential expression status
dif$DEG.status = "NS"

# if log2Foldchange > cutoff and pvalue < cutoff, set as "upregulated"
dif$DEG.status[(dif$log2FoldChange > cutoff.FC &
                 dif$padj < cutoff.pvalue)] = "upregulated"

# pvalue only
dif$DEG.status[(dif$log2FoldChange < -cutoff.FC &
                 dif$padj < cutoff.pvalue)] = "downregulated"

# add column to remove gene name unless its in the "gene" argument list
dif$genes.to.show = if_else(dif$symbol %in% genes, as.character(dif$symbol), '')
print(table(dif$genes.to.show))

# factor colors
myColors = c("blue", "gray50", "red")
names(myColors) = levels(factor(dif$DEG.status))
colScale = scale_color_manual(name="DEG.status", values=myColors)

# plot
p <- ggplot(data=dif[-log10(dif$padj) < ylim & (dif$log2FoldChange < xlim & dif$log2FoldChange > -xlim),],
            aes(x=log2FoldChange,
                y=-log10(padj),
                col=DEG.status)) +
  geom_point() +
  theme_classic()

# Add lines as before...
p2 <- p + ggtitle(title) +
  geom_vline(xintercept=c(-cutoff.FC, cutoff.FC), col="green") +
  geom_hline(yintercept=-log10(cutoff.pvalue), col="green") +
  colScale
```

```
  p3 <- p2 +
    geom_text_repel(aes(label=genes.to.show),
                    nudge_x = 5.0,
                    show.legend=F,
                    max.overlaps = Inf,
                    #arrow = arrow(length = unit(0.02, "npc")),
                    box.padding   = 0.5,
                    #point.padding = 0.5,
                    segment.color = 'black')

  if(label == TRUE) {
    print(grid.arrange(p2, p3, nrow=1))}
  else{
    print(p2)
  }
}



##############
### getDEG ###
##############
#'bad_weak_ptr'
#'In the context of R, this error often surfaces in packages that use C++ for performance-critical code, such as those in Bioconductor or other packages interfacing with external libraries. The R environment manages the lifecycle of objects, and mismatches or corruption in the management of these objects can lead to a bad_weak_ptr error.
#if (!requireNamespace("BiocManager", quietly = TRUE))
#  install.packages("BiocManager")

#BiocManager::install(version = "3.17")  # Ensure correct Bioconductor version
#BiocManager::install("org.Mm.eg.db", type = "source")  # Reinstall from source

#update.packages(ask = FALSE)  # Update all packages
```

```r
#BiocManager::install()  # Update Bioconductor packages

#rm(list = ls())  # Clear workspace
#sessionInfo()  # Check session info for conflicts

#if (!requireNamespace("org.Mm.eg.db", quietly = TRUE)) {
#  BiocManager::install("org.Mm.eg.db")
#}

library(org.Mm.eg.db)
ensembl_keys <- keys(org.Mm.eg.db, keytype = "ENSEMBL")
ensembl_keys
dim(ensembl_keys)

# must run DESeq() on dds first!
getDEG = function(dds, contrast){
  dif = as.data.frame(results(dds,
                              contrast = contrast))

  # order by fold change
  dif = dif %>%
    arrange(desc(log2FoldChange))

  # annotate symbols
  dif = dif %>%
    mutate(symbol = toupper(mapIds(org.Mm.eg.db,
                                   keys=row.names(dif),
                                   column="SYMBOL",
                                   keytype="ENSEMBL",
                                   multiVals="first")),
           .before=1) %>%
    mutate(ID = rownames(dif), .before=1) %>%
    filter(padj < 0.5)
```

```
  # filter out NA symbols
  dif.noNA = dif %>%
    filter(!is.na(symbol))

  # plot volcano
  p = EnhancedVolcano(dif.noNA,
                      lab = dif.noNA$symbol,
                      x = 'log2FoldChange',
                      y = 'padj',
                      pCutoff=0.05,
                      FCcutoff=0.58, # log2FC cutoff of 0.58 -> FC of 1.5
                      title=paste("DEG_", contrast[2], "_vs_", contrast[3],sep=""),
                      drawConnectors = T)
  #print(p)
  pdf(paste(format(Sys.Date(),"%m-%d-%y_"), "DEG_", contrast[2], "_vs_", contrast[3], "-lfc058_padj005.pdf",
sep=""),
      height=10,
      width=10)
  print(p)
  dev.off()

  # write output to file
  outfile=paste(format(Sys.Date(),"%m-%d-%y_"), "DEG_", contrast[2], "_vs_", contrast[3], "-
lfc058_padj005.txt", sep="")
  write_tsv(dif, outfile)

  return(dif)

}
```

Salmon and Heatmap Analysis

```
## R script for tximport of STAR/salmon counts
#Written by Jeremiah Holt
#Adapted by Minjeong Kim

# For murine samples (mm10, GRCm38.p4, GENCODE M25)
#library(readr)
library(tidyverse)
library(tximport)
library(DESeq2)

library(Mus.musculus) #OrganismDb object (OrganismDbi)
library(org.Mm.eg.db) #Org.db object (AnnotationDbi)
library(AnnotationDbi)
library(biomaRt)

#for Mac: ~/OneDrive - University of Tennessee/1_MakowskiLab/Scripts/rna_pipeline-main/scripts/mm10/R_functions_mm10.R
#for Windows(lab): C:/Users/mkim70/OneDrive - University of Tennessee/1_MakowskiLab/Scripts/~~

source("C:/Users/mkim70/OneDrive - University of Tennessee/1_MakowskiLab/Scripts/rna_pipeline-main/scripts/mm10/R_functions_mm10.R")

setwd("C:/Users/mkim70/OneDrive - University of Tennessee/1_MakowskiLab/1_FMT_Mg/FMT/")
getwd()

# get file names and extract samples from each
files = list.files(path="./", pattern="*\\.sf", recursive=T)
samples = gsub("_.*","", files)
samples = gsub(".*/", "", samples)
names(files) = samples
length(files) #12

# extract condition from sample-conditions file
```

```
conditionTable = read_tsv("C:/Users/mkim70/OneDrive - University of Tennessee/1_MakowskiLab/1_FMT_Mg/FMT/SampleTable/FMT_Sample_Conditions_v5.txt") %>%
  dplyr::rename(SampleID=`Sample Name`) %>%
  filter(SampleID %in% names(files))
dim(conditionTable) # 12 8

# check for missing samples
missing = conditionTable[which(!conditionTable$SampleID %in% names(files)),]

all(conditionTable$SampleID %in% names(files)) #TRUE
all(conditionTable$SampleID == names(files)) #TRUE
all(names(files) %in% conditionTable$SampleID) #TRUE

# make colData table for DESeq2
sampleTable = data.frame(SampleID=names(files),
                         file=files,
                         treatment=factor(conditionTable$`Treatment Group`),
                         size = factor(conditionTable$log2scaled_Tumor_volume_mm3))

dim(sampleTable) # 12 4
table(sampleTable$treatment)
table(sampleTable$size)

######################################
### Make TxDb object for tximport ###
######################################
library(GenomicFeatures)

# TxDb object
txdb = makeTxDbFromGFF("C:/Users/mkim70/OneDrive - University of Tennessee/1_MakowskiLab/Scripts/Annotations/gencode.vM25.annotation.gtf",
                       dataSource = "GENCODE.vM25", organism = "Mus musculus") #this takes long.
k = AnnotationDbi::keys(txdb, keytype = "TXNAME")
tx2gene = AnnotationDbi::select(txdb, k, "GENEID", "TXNAME")
```

```
tx2gene = tx2gene %>%
  mutate(TXNAME = gsub("\\..*", "", TXNAME), GENEID = gsub("\\..*", "", GENEID)) # remove version suffix
from transcripts and genes


write_tsv(tx2gene, "1206-23_tx2gene_gencode.vM25.txt") #ENST to ENSG matching table
colnames(tx2gene)  #[1] "TXNAME" "GENEID"


# merge txdb w/ OrganismDbi
library(Mus.musculus)
M.musculus = makeOrganismDbFromTxDb(txdb=txdb, keytype="ENSEMBLTRANS")
tx <- transcriptsBy(M.musculus, "gene",
                    columns = "ENSEMBL")


# add gene symbols
tx2gene2symbol = tx2gene %>%
  dplyr::rename(ENSEMBLTRANS=TXNAME) %>%
  left_join(., select(org.Mm.eg.db, keys(org.Mm.eg.db, keytype="ENSEMBLTRANS"), "SYMBOL",
"ENSEMBLTRANS"))


dim(tx2gene2symbol) #143032   3
head(tx2gene2symbol)
colnames(tx2gene2symbol) #[1] "ENSEMBLTRANS" "GENEID" - ENSG      "SYMBOL" - not sure what kind of
symbol it is.


##################################
### Read files using tximport ###
##################################


all(file.exists(files)) #TRUE
txi = tximport(files,
               type="salmon",
               tx2gene=tx2gene,
               ignoreTxVersion=T)
names(txi) #[1] "abundance"          "counts"             "length"             "countsFromAbundance"
```

```
# transcript per million (TPM) matrix
counts.matrix = txi$counts
dim(counts.matrix) # 48440    20
                   # 47880    13 (vM25)

counts.df = as.data.frame(counts.matrix) %>%
  mutate(GeneID = rownames(.), .before=1)
dim(counts.df) #47880 13 (vM25)
head(counts.df)
write_tsv(counts.df, "1206-23_n13_TPMcounts_matrix.txt")

########################
### Plug into DESeq2 ###
########################

ddsTxi = DESeqDataSetFromTximport(txi,
                                  colData = sampleTable,
                                  #design = ~ class.label.km4
                                  #design = ~ group + class.label.km4
                                  #design = ~ group
                                  design = ~ treatment # use this when normals are included w/ blind = F
)
dim(ddsTxi) # 48440    20
            # 47880    20 (vM25)
            # 47880    12 (vM25)

head(counts(ddsTxi))

# pre filter for genes that dont have at least 10 total counts
keep = rowSums(counts(ddsTxi) >= 10) >= 3
dds = ddsTxi[keep,]
dim(dds) #20247    20
```

```
        #[1] 20065    20 (vM25)
        #[1] 18002    14
        #[1] 17784    13
        #[1] 17508    12

# get Biotype information for each gene using biomaRt
sensembl = biomaRt::useMart("ensembl",
                            dataset="mmusculus_gene_ensembl",
                            host="https://www.ensembl.org")

goids.mm10 = biomaRt::getBM(attributes = c('ensembl_gene_id', 'gene_biotype'),
                            filters = 'ensembl_gene_id',
                            values = rownames(dds),
                            mart = sensembl)

table(goids.mm10$gene_biotype)
colnames(goids.mm10)
head(goids.mm10)
write_tsv(goids.mm10, "1206-23_goidsmm10vM25.txt")

IDsToKeep = goids.mm10 %>%
  dplyr::filter(gene_biotype == "protein_coding")  # keep protein coding
dim(IDsToKeep) #15985     2
            #[1] 16026     2 (vM25)
            #[1] 15113     2
            #[1] 14996     2
            #[1] 14787     2
write_tsv(IDsToKeep,"1206-23_14787pcg_n12_IDsToKeep.txt")

# filter genes and remove controls
dds = dds[IDsToKeep$ensembl_gene_id,]
dim(dds) #15985    20
         #[1] 16026    20 (vM25)
```

```
        #[1] 15113    14
        #[1] 14996    13
        #[1] 14787    12
# check meta data
colData(dds)

# run DESeq
dds = DESeq(dds)

# check distributions with counts
boxplot(counts(dds)+1)
# check distributions after log2 transformation
boxplot(log2(counts(dds)+1))
boxplot(log2(counts(dds, normalized=T)+1))

Log2counts <- log2(counts(dds))
boxplot(Log2counts, las = 2,
        cex.axis = 0.7,
        ylab = "log2(read count)",
        main = "log2-scaled read counts [not normalized]")
normLog2counts <- log2(counts(dds, normalized = T)+1)
boxplot(normLog2counts, las =2,
        cex.axis = 0.7,
        ylab = "log2(normalized read count)",
        main = "log2-scaled normalized read counts")
dev.off()

# create TPM matrix with mouse ENSG ID
TPM.matrix = as.data.frame(txi$abundance[IDsToKeep$ensembl_gene_id,]) %>%
  mutate(symbol = mapIds(org.Mm.eg.db,
                         keys=row.names(.),
                         column="SYMBOL",
                         keytype="ENSEMBL",
```

```
                    multiVals = "first"),
         .before = 1) %>%
  mutate(ID = row.names(.), .before = 1)
dim(TPM.matrix) #15985    22
                #[1] 16026    22 (vM25)
                #[1] 15113    16
                #[1] 14996    15
                #[1] 14787    14
view(TPM.matrix)
head(TPM.matrix)
class(TPM.matrix)
write_tsv(TPM.matrix, "1206-23_pcg14787-n12_TPM_wENSG_GeneSymbol.txt")



# get DEGs
dif <- getDEG(dds, c("treatment", "aPD1", "IgG")) %>%
  mutate(UP.DOWN = if_else(log2FoldChange >= 0, 1, 0))

# regularized log normalization (NORMALIZATION FOR DOWNSTREAM ANALYSIS AND VIZUALIZATION)
rlog = rlog(dds, blind=F) # blind = T if study design shouldn't be used to normalize (use blind = F w/ batches/backgrounds, blind = T for unbiased QC/PCA/clustering)
head(assay(rlog))
results = as.data.frame(assay(rlog))
write_tsv(results, "1206-23_rlog_readcount.txt")

view(results)
dim(results) #15146    12
              #[1] 16026    20 (vM25)
              #[1] 15113    14
              #[1] 14996    13
              #[1] 14787    12
results = results %>%
  mutate(GeneSymbol = (mapIds(org.Ms.eg.db,
                       keys=row.names(counts(dds, normalized=T)),
```

```
                        column="SYMBOL",
                        keytype="ENSEMBL",
                        multiVals="first")), .before=1) %>%
  mutate(GeneID = rownames(results), .before=1)
dim(results) #14996 13
        #[1] 14787    12
view(results)
rownames(results)
colnames(results)
write.table(results, "1206-23_rlog_readcount_wENBG.txt", sep = "\t")
#and then open file, move 1st cell into the right, and add "ID"


# merge TPM counts with gene symbol
dim(results)
view(results)


#open "date_normalized_rlog.txt" and move 1st row to the right and add "ID" header at (1,1). Save it and recall it again.
rlognormcount <- read.table ("1206-23_rlog_readcount_wENBG.txt", sep="\t", header = T)
view(rlognormcount)
colnames(rlognormcount)


DEGlist1 <- read.table("1206-23_DEG_aPD1_vs_IgG-lfc058_padj005.txt", sep ="\t", header = T)
rownames(DEGlist1)
colnames(DEGlist1)  #[1] "ID"            "symbol"        "baseMean"       "log2FoldChange" "lfcSE"          "stat"
"pvalue"        "padj"


print(colnames(DEGlist1))
print(colnames(rlognormcount))
DEG_rlognormcount <-  merge(rlognormcount, DEGlist1, by.x="ID", by.y = "ID")
colnames(DEG_rlognormcount)
dim(DEG_rlognormcount) #412 21 (vM25, n=13) 11-28-2023
              #[1] 487  20 (vM25, n=12) 12-06-2023
write_tsv(DEG_rlognormcount, "1206-23_rlognormcount_of_DEG.txt")
```

```
###################################################################################
# normalize for downstream analysis, e.g. clustering (not DEG)                #
# use VST over rlog for sample n > 30                                          #
###################################################################################
install.packages("colorRamp2")
library(tidyverse)
library(DESeq2)
library(colorRamp2)
# Variance stabilizing transformation (VST) or Regularized log transformation (RLOG) form DESeq2


# about "blind" argument from DESeq2 manual: logical, whether to blind the transformation to the experimental design.
# blind=TRUE should be used for comparing samples in an manner unbiased by prior information on samples, for example to perform sample QA (quality assurance).
# blind=FALSE should be used for transforming data for downstream analysis, where the full use of the design information should be made.
# blind=FALSE will skip re-estimation of the dispersion trend, if this has already been calculated.
# If many of genes have large differences in counts due to the experimental design, it is important to set blind=FALSE for downstream analysis.


rlg = rlog(dds, blind=T) # blind = F here because different cell line backgrounds were used in this experiment
head(assay(rlg))
results = as.data.frame(assay(rlog))
dim(results) #16026 20
        #[1] 15113    14
        #[1] 14996    13
        #[1] 14787    12
results %>%
  mutate(GeneSymbol = (mapIds(org.Mm.eg.db,
                    keys=row.names(counts(dds, normalized=T)),
                    column="SYMBOL",
                    keytype="ENSEMBL",
                    multiVals="first")), .before=1) %>%
  mutate(GeneID = rownames(results), .before=1) %>%
```

```
  write_tsv("1206-23_pcg14787-n12_salmon_mm10_counts_RLOG_normalized_log2_DESeq2.txt")


# read in results and convert to matrix

results = read_tsv("1206-23_pcg14787-n12_salmon_mm10_counts_RLOG_normalized_log2_DESeq2.txt")

dim(results)      # 16026  22  | w/ 2 gene label columns (GeneID and GeneSymbol) - vM25

                # 15113 16

                # 14996 15

                #[1] 14787    14 (n=12)

colnames(results)  # [1] "GeneID"            "GeneSymbol"        "S000005-38108-002" "S000005-38109-002"
"S000005-38110-002" "S000005-38111-002" "S000005-38113-002"

                 #[8] "S000005-38114-002" "S000005-38115-002" "S000005-38116-002" "S000005-38118-002"
"S000005-38119-002" "S000005-38120-002" "S000005-38121-002"

                 #[15] "S000005-38124-002" "S000005-38126-002"



results.matrix = data.matrix(results[,-c(1:2)]) # remove geneID columns #[,-c(1:2)] removes first two columns.

head(results)

rownames(results.matrix) = results$GeneID

head(results.matrix)

dim(results.matrix) #16026 20

                  #[1] 15113    14

                  #[1] 14996    13

                  #[1] 14787    12

################################

### Visualize distributions ###

################################


#### this uses funcitons from R_functions.R


# plot and compare

boxplot(log2(TPM.matrix[,-c(1,2)] + 1), xlab="Sample", ylab = "TPM pseudocounts (log2+1)", main = "TPM for
protein coding mRNA only (n=18558)")

boxplot(log2(TPM.matrix[,-c(1,2)] + 1), xlab="Sample", ylab = "TPM pseudocounts (log2+1)", main = "TPM for
all measured RNA (n=29422)")
```

```
boxplot(log2(counts(dds, normalized=F)+1), xlab ="Sample", ylab="Raw pseudocounts (log2+1)")

boxplot(results[,-c(1,2)], xlab ="Sample", ylab="Log2 normalized counts (RLOG)")

boxplot(log2(counts(dds, normalized=T)+1), xlab ="Sample", ylab="Library sized normalized pseudocounts (log2+1)")


source("C:/Users/mkim70/OneDrive - University of Tennessee/1_MakowskiLab/Scripts/rna_pipeline-main/scripts/R_functions.R")

# call plotting functions from 2_R_functions.R

p = plotDistribution(dds,

                  plot_type="boxplot",

                  norm=F)

print(p + ggtitle("Pseudocounts: log2(count+1)"))


p = plotDistribution(dds,

                  plot_type="boxplot",

                  norm=T)

print(p + ggtitle("Library Size Normalized Psuedocounts: log2(TPM+1)"))


# density

p = plotDistribution(dds,

                  plot_type="density",

                  norm=F)

print(p + ggtitle("Psuedocounts: log2(TPM+1)"))


p = plotDistribution(dds,

                  plot_type="density",

                  norm=T)

print(p + ggtitle("Library Size Normalized Psuedocounts: log2(TPM+1)"))


# normalized

p = plotDistributionNorm(rlg, plot_type="boxplot")

print(p + ggtitle("RLOG Normalized Expression"))


p = plotDistributionNorm(rlg, plot_type="density")
```

```
print(p + ggtitle("RLOG Normalized Expression"))



################################
### PCA plots and clusters ###
################################

#### this uses built in PCA plotting functions from DESeq2

# built in DESeq2 functions    #vsd -> rlg if you use rlog normalization.
#plotPCA(vsd, intgroup=c("treatment"), ntop=1000)
#plotPCA(vsd, intgroup=c("tissue"), ntop=1000)
#plotPCA(vsd, intgroup=c("background"), ntop=1000)

plotPCA(rlg, intgroup=c("treatment"), ntop=1000)
se <- SummarizedExperiment(log2(counts(dds, normalized = TRUE) +1),
                           colData = colData(dds))
plotPCA( DESeqTransform(se), intgroup = c("treatment"), ntop = 500)




###########################################
### Get quick heatmaps for MAD genes ###
###########################################

###########################################
library(ComplexHeatmap)
library(circlize)

# this is custom function for making heatmaps from top 25% of genes based on median absolute deviation (MAD)

# Now, calculate the minimum and maximum values - Size is log2-scaled tumor size(mm3)
min_size = min(sampleTable$size, na.rm = TRUE)
```

```
max_size = max(sampleTable$size, na.rm = TRUE)

#Change sampleTable$size from factor to Numeric.
# Convert 'Size' column to numeric
sampleTable$size <- as.numeric(as.character(sampleTable$size))

# Check the data type of the 'size' column
class(sampleTable$size)

# Get indices of 'IgG' and 'aPD1' samples
igG_indices <- which(sampleTable$treatment == "IgG")
aPD1_indices <- which(sampleTable$treatment == "aPD1")

# Combine indices, putting 'IgG' samples first followed by 'aPD1' samples
combined_indices <- c(igG_indices, aPD1_indices)

# Calculate Log2FC between 'aPD1' and 'IgG' samples
aPD1_indices <- which(sampleTable$treatment == "aPD1")
IgG_indices <- which(sampleTable$treatment == "IgG")
aPD1_samples <- dds[, aPD1_indices, drop = FALSE] # Ensure drop = FALSE to keep as a matrix/data frame
IgG_samples <- dds[, IgG_indices, drop = FALSE]
dim(IgG_samples)    #[1] 14996     8
dim(aPD1_samples)   #[1] 14996     5

##########################################################################################
##############################
library(ComplexHeatmap)
library(circlize)
library(colorRamp2)

# this is custom function for making heatmaps from top 25% of genes based on median absolute deviation
(MAD)
sampleTable$size
class(sampleTable$size)
```

```
#simple heatmap function
mad.heatmap = function(matrix, sampleTable, name){

  # filtered MAD
  mad = apply(matrix,1,mad)
  mad.cutoff= quantile(mad, 0.75)  # if you want to show top 25% of genes, than the quantile should be 0.75
  mad.subset = matrix[which(mad>mad.cutoff),]
  #mad.subset = matrix[dif$ID, ]
  dim(mad.subset) #4427   25



  # scale
  mad.matrix.scaled = t(scale(t(data.matrix(mad.subset))))
  rownames(mad.matrix.scaled) = rownames(mad.subset)
  head(mad.matrix.scaled)
  dim(mad.matrix.scaled) #4427   25

  matrix.scaled = t(scale(t(matrix)))

  #heatmap color function
  col_fun = colorRamp2(c(-2, 0, 2), c("dodgerblue3", "ivory", "firebrick1"))

  all(sampleTable$sample == colnames(mad.matrix.scaled))

  # heatmaps
  ha = HeatmapAnnotation( Treatment = sampleTable$treatment,
                          Size = sampleTable$size,
                          IFNG = matrix.scaled["ENSMUSG00000055170",], #IFNG and PRKCD are not in
mad.matrix.scaled.
                          PRKCD = matrix.scaled["ENSMUSG00000021948",],
                          PDCD1 = matrix.scaled["ENSMUSG00000026285",],
                          PTPRC = matrix.scaled["ENSMUSG00000026395",],
                          CD8A = matrix.scaled["ENSMUSG00000053977", ],
```

```
                    CD4 = matrix.scaled["ENSMUSG00000023274", ],
                    CD3E = matrix.scaled["ENSMUSG00000032093", ],
                    CD3D = matrix.scaled["ENSMUSG00000032094", ],
                    col=list(
                      IFNG = col_fun,
                      PRKCD = col_fun,
                      PDCD1 = col_fun,
                      PTPRC = col_fun,
                      CD8A = col_fun,
                      CD4 = col_fun,
                      CD3E = col_fun,
                      CD3D = col_fun,
                      Treatment = c( "IgG" = "dodgerblue4", "aPD1" = "firebrick"),
                    Size = colorRamp2(
                      c(min_size, max_size),
                      c("lightgoldenrod1", "lightsalmon3")
                    )),
                simple_anno_size = unit(0.75, "cm"),
                simple_anno_size_adjust = TRUE,
                  annotation_name_side = "right",
                  show_legend = c(rep(TRUE, 2), rep(FALSE, 6))
)


  hm = Heatmap(mad.matrix.scaled,
          col = col_fun,
          column_title = paste("All samples n=", ncol(mad.matrix.scaled), sep=""),
          #row_title = paste("Differentially Expressed Genes n=", nrow(mad.matrix.scaled), sep=""),
          row_title = paste("Top 25% most variable genes n=", nrow(mad.matrix.scaled), sep = ""),
          show_row_names = F,
          cluster_rows = T, # should clustering be done on rows?
          clustering_method_rows = "complete",
          row_km = 3,
```

```
                row_km_repeats = 100,
                row_gap = unit(2, "mm"),
                #row_split = dif$UP.DOWN,
                column_split=sampleTable$treatment, # force split then cluster
                cluster_columns =T, # should clustering be done on columns? Yes. (MJ)
                clustering_method_columns = "complete",
                #column_order = cluster_order,
                #column_km = 4,
                #column_km_repeats = 100,
                column_gap = unit(2, "mm"),
                border = FALSE,
                top_annotation = ha,
                heatmap_legend_param = list(col = col_fun,
                                  title = "Scaled Gene Exp."
                                  #direction = "horizontal"
                )
  )

  pdf(paste(name,".pdf", sep=""), width = 14, height = 14)
  draw(hm, heatmap_legend_side = "right", annotation_legend_side = "right", merge_legend=TRUE)
  dev.off()
}



# call function that filters MAD and then scales / draw heatmap and save results with retained order.
mad.heatmap(results.matrix, sampleTable,
          "1211-23_split_km_pdg14787_n12_top25_MAD_heatmap_7-extractTest")

#ext <- mad.matrix.scaled[rowOrder, columnOrder]
r_list = row_order(hm)
r_list          #clustered by k-means but why it's not list? It's list!
c_list = column_order(hm)
c_list
```

```
rowOrder = unlist(r_list)

columnOrder = unlist(c_list)

geneOrder = as.data.frame(rowOrder)

write_tsv(geneOrder, "geneOrder.txt")


ext <- as.data.frame(results.matrix[rowOrder, columnOrder]) #maybe I should change input matrix? mad.matrix.scaled is not found.

write_tsv(ext, file = "1211-23_madmatrixscaled_order_retained2.txt")  #gene ID has gone. not usable.



#in case of you fed them with DEGs,

mad.heatmap(results.matrix, sampleTable,

        "1206-23_split_DEG_n12_MAD_heatmap_1")
```
